# Distinct Roles of Hand2 in Initiating Polarity and Posterior *Shh* Expression during the Onset of Mouse Limb Bud Development

**DOI:** 10.1371/journal.pgen.1000901

**Published:** 2010-04-08

**Authors:** Antonella Galli, Dimitri Robay, Marco Osterwalder, Xiaozhong Bao, Jean-Denis Bénazet, Muhammad Tariq, Renato Paro, Susan Mackem, Rolf Zeller

**Affiliations:** 1 Developmental Genetics, Department of Biomedicine, University of Basel, Basel, Switzerland; 2 Cancer and Developmental Biology Laboratory, National Cancer Institute, Bethesda, Maryland, United States of America; 3 Department of Biosystems Science and Engineering, ETH Zurich, Basel, Switzerland; 4 Faculty of Sciences, University of Basel, Basel, Switzerland; Harvard Medical School, United States of America

## Abstract

The polarization of nascent embryonic fields and the endowment of cells with organizer properties are key to initiation of vertebrate organogenesis. One such event is antero-posterior (AP) polarization of early limb buds and activation of morphogenetic Sonic Hedgehog (SHH) signaling in the posterior mesenchyme, which in turn promotes outgrowth and specifies the pentadactylous autopod. Inactivation of the *Hand2* transcriptional regulator from the onset of mouse forelimb bud development disrupts establishment of posterior identity and *Shh* expression, which results in a skeletal phenotype identical to *Shh* deficient limb buds. In wild-type limb buds, Hand2 is part of the protein complexes containing Hoxd13, another essential regulator of *Shh* activation in limb buds. Chromatin immunoprecipitation shows that Hand2-containing chromatin complexes are bound to the far upstream *cis*-regulatory region (ZRS), which is specifically required for *Shh* expression in the limb bud. Cell-biochemical studies indicate that Hand2 and Hoxd13 can efficiently transactivate gene expression via the ZRS, while the Gli3 repressor isoform interferes with this positive transcriptional regulation. Indeed, analysis of mouse forelimb buds lacking both *Hand2* and *Gli3* reveals the complete absence of antero-posterior (AP) polarity along the entire proximo-distal axis and extreme digit polydactyly without AP identities. Our study uncovers essential components of the transcriptional machinery and key interactions that set-up limb bud asymmetry upstream of establishing the SHH signaling limb bud organizer.

## Author Summary

During early limb bud development, posterior mesenchymal cells are selected to express *Sonic Hedgehog* (*Shh*), which controls antero-posterior (AP) limb axis formation (axis from thumb to little finger). We generated a conditional loss-of-function *Hand2* allele to inactivate *Hand2* specifically in mouse limb buds. This genetic analysis reveals the pivotal role of Hand2 in setting up limb bud asymmetry as initiation of posterior identity and establishment of the *Shh* expression domain are completely disrupted in *Hand2* deficient limb buds. The resulting loss of the ulna and digits mirror the skeletal malformations observed in *Shh*-deficient limbs. We show that Hand2 is part of the chromatin complexes that are bound to the *cis*-regulatory region that controls *Shh* expression specifically in limb buds. In addition, we show that Hand2 is part of a protein complex containing Hoxd13, which also participates in limb bud mesenchymal activation of *Shh* expression. Indeed, Hand2 and Hoxd13 stimulate ZRS–mediated transactivation in cells, while the Gli3 repressor form (Gli3R) interferes with this up-regulation. Interestingly, limb buds lacking both *Hand2* and *Gli3* lack AP asymmetry and are severely polydactylous. Molecular analysis reveals some of the key interactions and hierarchies that govern establishment of AP limb asymmetries upstream of SHH.

## Introduction

An important step during the initiation of vertebrate organogenesis is the setting-up of morphogenetic signaling centers that coordinately control cell specification and proliferation. One paradigm model to study these processes is the developing limb bud and recent studies have revealed how morphogenetic Sonic hedgehog (SHH) signaling from the zone of polarizing activity (ZPA) and Fibroblast growth factor (FGF) signaling from the apical ectodermal ridge (AER) coordinate cell specification with proliferation along both major limb bud axes [1]. AER-FGF signaling mainly controls the establishment of the proximo-distal (PD) limb bud axis (sequence: stylopod-zeugopod-autopod) [2], while SHH signaling by the polarizing region controls antero-posterior (AP) axis formation (radius and ulna, thumb to little finger) [3],[4]. Cells receiving the SHH signal inhibit the constitutive processing of Gli3 to its repressor form (Gli3R) and upregulate the expression of the Gli1 transcriptional activator, which results in positive regulation of SHH target genes [5]–[7]. In limb buds of mouse embryos lacking *Gli3*, the expression of initially posteriorly restricted genes such as *Hand2*, *5′HoxD* genes and the BMP antagonist *Gremlin1* (*Grem1*) expands anteriorly from early stages onwards and an anterior ectopic *Shh* expression domain is established at late stages [8]. However, the resulting digit polydactyly arises in a SHH-independent manner, as limbs of embryos lacking both *Shh* and *Gli3* are morphologically and molecularly identical to *Gli3* deficient mouse embryos [9],[10]. These and other studies indicate that Gli3 acts initially up-stream of SHH signaling to restrict the expression of genes activated prior to *Shh* to the posterior limb bud [11] and that SHH-mediated inhibition of Gli3R production is subsequently required to enable distal progression of limb bud development [9].

The molecular interactions that polarize the nascent limb bud along its AP axis and activate SHH signaling in the posterior limb bud mesenchyme have only been partially identified. Previous studies implicated the basic helix-loop-helix (bHLH) transcription factor *Hand2* (*dHand*) in these early determinative processes upstream of SHH signaling [1]. In particular, the development of fin and limb buds of *Hand2* deficient mouse and zebrafish embryos arrests at an early stage and no *Shh* expression is detected [12],[13]. This early developmental arrest in conjunction with massive generalized apoptosis of *Hand2* deficient mouse limb buds precluded an *in depth* analysis of the molecular circuits and signaling systems that control initiation and progression of limb bud development. Furthermore, transgene-mediated over-expression of *Hand2* induces digit duplications in mouse limb buds [14]. The functional importance of Hand2 as a transcriptional regulator in these processes was further corroborated by an engineered mutation that inactivates the Hand2 DNA binding domain in mouse embryos, which results in limb bud defects resembling the *Hand2* null phenotype [15]. Cell-biochemical analysis showed that Hand2 interacts with so-called *Ebox* DNA sequence elements most likely as a heterodimer with other bHLH transcription factors such as E12 [16],[17] and Twist1, which is also required for early limb bud development [18],[19].

Genetic analysis in mouse embryos showed that *Gli3* is required to restrict *Hand2* expression to the posterior limb bud mesenchyme as part of a mutually antagonistic interaction [11]. This interaction was proposed to pre-pattern the limb bud mesenchyme along its AP axis prior to activation of SHH signaling. However, the functional importance of this pre-patterning mechanism for normal progression of limb development remained unknown. Additional pathways are also required for establishment of the *Shh* expression domain in the posterior limb bud mesenchyme such as retinoic acid signaling from the flank and AER-FGF8 signaling [20],[21]. During the onset of limb bud development, the expression of the 5′ most members of the *HoxD* gene cluster is restricted to the posterior mesenchyme by *Gli3* [22],[23]. During these early stages, the 5′HoxA and 5′HoxD transcriptional regulators are required to activate *Shh* expression in the posterior limb bud mesenchyme [24]–[26]. Consistent with this genetic analysis, the Hoxd10 and Hoxd13 proteins interact directly with the *cis*-regulatory region that controls *Shh* expression in limb buds [27]. This evolutionary conserved *cis*-regulatory region is called ZPA regulatory sequence (ZRS) and is located about 800 Kb up-stream of the *Shh* gene [28]. Genetic inactivation of the highly conserved core region of the ZRS (termed MFCS1) results in limb bud-specific loss of *Shh* expression and a *Shh* loss-of-function limb skeletal phenotype [29]. Interestingly, this limb bud specific *cis*-regulatory region is absent from vertebrate species that have lost their limbs during evolution [30]. Transgenic analysis in mouse embryos revealed that ZRS-*LacZ* transgenes recapitulate major aspects of *Shh* expression in limb buds [28]. However, this study did not reveal specific *cis*-regulatory elements or sub-regions within the ZRS that regulate transcription, but rather indicated that the entire ZRS is required for correct *Shh* expression. A recent study shows that the ZRS interacts directly with the *Shh* transcription unit in both the anterior and posterior limb bud mesenchyme [31]. However, the *Shh* locus loops out of its chromosomal territory only in the posterior mesenchyme, which results in initiation of transcription. The evolutionary conserved function of the ZRS is underscored by an ever increasing large number of point mutations that are scattered through large parts of ZRS region and cause congenital preaxial polydactylies (PPD) in humans and many other mammals [32]. In summary, these studies establish that the far upstream ZRS *cis*-regulatory region controls *Shh* expression in different tetrapod species and that point mutations cause PPD, while deletion of the central part of the ZRS results in limbless phenotypes.

We have generated a conditional *Hand2* mouse loss-of-function allele and use it to study the requirement of *Hand2* during limb bud initiation. Inactivation of *Hand2* in the forelimb field mesenchyme using the *Prx1*-Cre transgenic mouse strain disrupts the development of posterior skeletal elements. Complete and early inactivation results in a limb skeletal phenotype identical to limbs lacking *Shh*. Indeed, establishment of the *Shh* expression domain in the posterior limb bud is disrupted and early molecular markers of posterior identity are lost, while anterior markers expand posteriorly. This reveals the early requirement of Hand2 for establishing posterior identity and activation of *Shh* expression. Using specific antibodies, we identify protein complexes containing both Hand2 and Hoxd13 transcriptional regulators in wild-type limb buds. Chromatin immunoprecipitation using Hand2 antibodies reveals the specific enrichment of the ZRS in comparison to adjacent non-ZRS DNA sequences in wild-type limb buds. Functional analysis of the DNA-protein interactions in cultured fibroblasts reveals that Hand2 and Hoxd13 transactivate expression of a ZRS-luciferase reporter construct, while this is partially inhibited by Gli3R, which has been previously shown to interact with 5′Hoxd proteins [33]. Indeed, mouse limb buds deficient for both *Gli3* and *Hand2* lack AP asymmetry along the entire PD limb axis and display severe digit polydactyly with complete loss of identities. Our study uncovers the interactions of Hand2 with the Gli3 and Hoxd13 transcriptional regulators and the far-upstream ZRS *cis*-regulatory region that are required to polarize the nascent limb bud mesenchyme and establish *Shh* expression in the posterior limb bud.

## Results

### Limb bud–specific inactivation of *Hand2* results in skeletal defects identical to *Shh* deficient limbs

**Figure 1:**
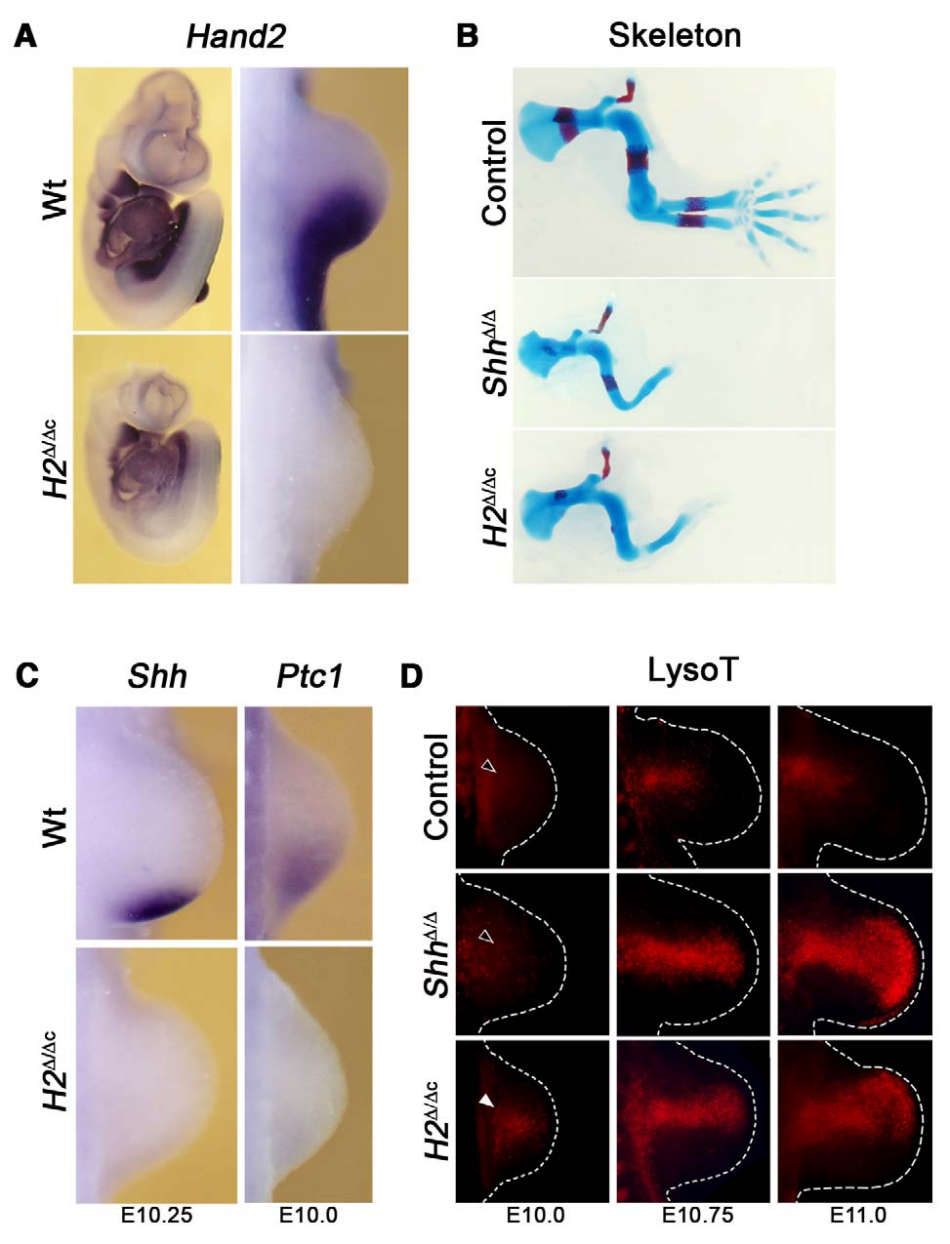
Early deletion of *Hand2* in mouse forelimb buds phenocopies the *Shh* loss-of-function skeletal phenotype. (A) Whole mount *in situ* hybridization detects *Hand2* transcripts in wild-type (Wt) and mouse embryos that lack the *Hand2* gene in their forelimb bud mesenchyme (*H2* ^Δ/Δc^) at E9.75 (28 somites). *Hand2* transcripts are absent from forelimb buds of *H2* ^Δ/Δc^ mouse embryos. (B) Skeletons of mouse forelimbs at E14.5, stained with alcian blue (cartilage) and alizarin red (bone). *Prx1*-Cre mediated inactivation of *Hand2* (*H2* ^Δ/Δc^) phenocopies the *Shh* ^Δ/Δ^ limb skeletal phenotype. Control: *Prx1-Cre* ^tg/+^. (C) *Shh* and *Ptc1* transcripts are absent from *H2* ^Δ/Δc^ limb buds at E10.25 (32 somites for *Shh*) and E10.0 (29 somites for *Ptc1*). (D) Detection of apoptotic cells by LysoTracker Red (LysoT). *Hand2* deficient limb buds are compared to control (*Prx1-Cre* ^tg/+^ and *H2* ^+/f^) and *Shh* ^Δ/Δ^ limb buds at E10.0 (30 somites), E10.75 (37 somites), and E11.0. The white arrowhead points to the precocious initiation of cell death in *H2* ^Δ/Δc^ forelimb buds (compare white to open arrowheads; n = 2/4). In all panels, limb buds are oriented with the anterior to the top and the posterior to the bottom.

Mouse embryos lacking *Hand2* die during mid-gestation due to cardiovascular defects and limb bud development arrests prior to formation of limb skeletal elements [12],[34]. Therefore, we generated a conditional *Hand2* loss-of-function allele by inserting two *loxP* sites into the locus (“floxed” allele: *Hand2* ^f^ or *H2* ^f^), which enables Cre-recombinase mediated deletion of the *Hand2* transcription unit (Figure S1). *Hand2* was inactivated in the limb bud mesenchyme (*H2* ^Δ ˜Δc^; Δc: conditional inactivation of the *Hand2* ^f^ allele) using the *Prx1*-Cre transgene, which is expressed in the forelimb field mesenchyme from about E8.5 onwards (14 somites) [35],[36]. The inactivation of *Hand2* was verified by monitoring the clearance of *Hand2* transcripts and proteins in forelimb buds and mesenchymal cells (Figure 1A and Figure S2A, S2B, S2C). Limb bud specific inactivation of *Hand2* (*H2* ^Δ ˜Δc^; Figure 1A) causes distal truncations of the forelimb skeleton and loss of the autopod (Figure 1B). The skeletal phenotypes of *Hand2* deficient forelimbs are variable, but the most severely affected cases (39% of all limbs, n = 80; Figure S3A, S3D) are identical to *Shh* deficient limbs (Figure 1B). Indeed, *Shh* expression and SHH signal transduction are lacking from a similar fraction of all *H2* ^Δ ˜Δc^ limb buds (Figure 1C and Figure S3C). Therefore, the most severely affected *H2* ^Δ ˜Δc^ limb buds correspond to the limb-specific complete *Hand2* loss-of-function phenotype (Figure 1A–1C and Figure S3). Between two and four digits form in hypomorphic *H2* ^Δ ˜Δc^ limbs (Figure S3A, S3D) as a likely consequence of residual *Hand2* expression, which triggers SHH signal transduction (Figure S3B, S3C).

In the most severely affected forelimb buds, cells along the entire PD axis, but in particular in the distal-anterior mesenchyme are eliminated by apoptosis (Figure 1D), which is distinct from the generalized apoptosis and developmental arrest of mouse embryos lacking *Hand2* constitutively (Figure S1D, S1E) [12]. In *H2* ^Δ ˜Δc^ forelimb buds, cell death is limited to the core mesenchyme at embryonic day E10.0 (Figure 1D, white arrowhead). In contrast, no significant apoptosis is detected in forelimb buds of wild-type and *Shh* deficient limb buds at these early stages (Figure 1D, open arrowhead). Therefore, *Hand2* is required for cell survival upstream of its role in activation of SHH signaling (Figure 1D, left panels). During progression of limb bud development, the apoptotic domain expands distal-anterior in *H2* ^Δ ˜Δc^ limb buds and becomes similar to the cell death domain observed in *Shh* deficient limb buds (Figure 1D, middle and right panels).

**Figure 2:**
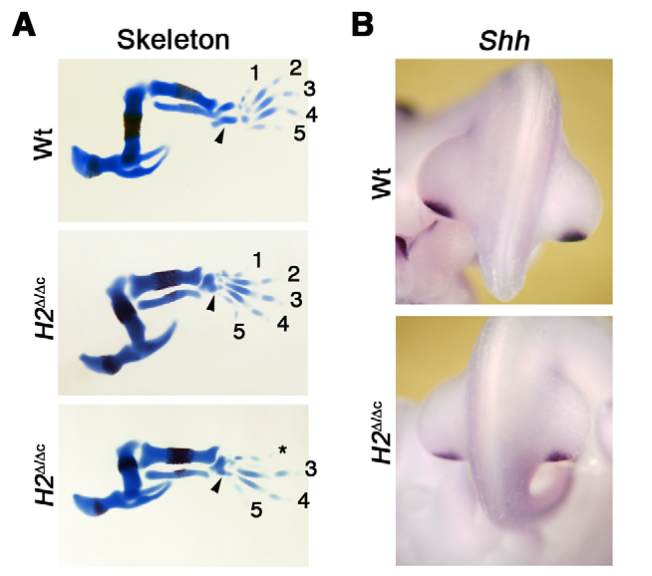
Delayed inactivation of *Hand2* in hindlimb buds results in rather normal *Shh* expression and development. (A) Hindlimb buds skeletons at E14.5, stained with alcian blue (cartilage) and alizarin red (bone). *Prx1*-Cre mediated inactivation of *Hand2* (*H2* ^Δ/Δc^) in hindlimb buds results in all cases in fusion of the tarsals (arrowheads) and formation of 5 (n = 11/24) or 4 (n = 13/24) digits. Please note that in latter case the formation of digit 2 and/or 3 (not shown), which depend mostly on long-range SHH signaling [7] is always affected. (B) *Shh* expression in wild-type and *Hand2* deficient hindlimb buds at E10.75 (37 somites). Note that the expression domain is correctly positioned in *H2* ^Δ/Δc^ hindlimb buds, but expression levels are reduced.

In mouse embryos, hindlimb development is delayed by ∼12 hrs and activation of the *Prx1*-Cre transgene in the posterior mesenchyme is delayed by ∼24 hrs in comparison to forelimb buds [35],[36]. The resulting ∼12 hrs delay in *Hand2* inactivation at equivalent stages in the posterior hindlimb bud allows formation of an autopod with 4–5 digits, while the tarsal bones are always fused (Figure 2A). Furthermore, inactivation of *Hand2* specifically in the distal forelimb bud mesenchyme from E10.5 onwards no longer alters skeletal development (data not shown). In agreement with the subtle skeletal alterations following *Prx1*-Cre-mediated *Hand2* inactivation in hindlimb buds (Figure 2A) *Shh* remains expressed, albeit at slightly lower levels than in wild-types (Figure 2B). Taken together, these studies show that Hand2 is essential to establish *Shh* expression in the posterior mesenchyme during initiation of limb bud development. Subsequently, it contributes to transcriptional up-regulation of *Shh* expression.

### Hand2 is essential for establishment of posterior identity upstream of SHH signaling

**Figure 3:**
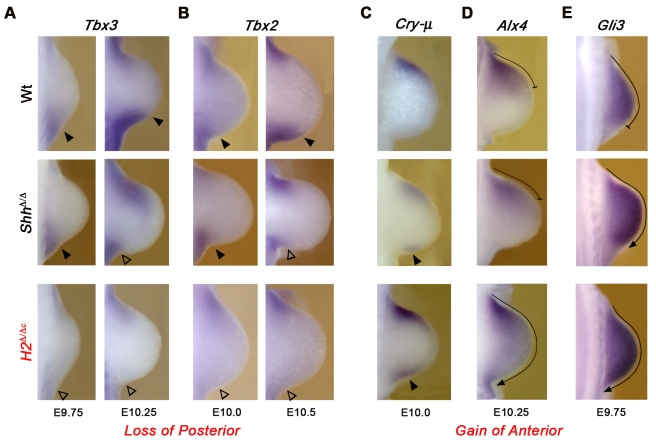
Establishment of posterior forelimb bud identity requires *Hand2*. (A,B) The loss of the posterior *Tbx3* and *Tbx2* expression domains in early *Hand2* deficient (*H2* ^Δ/Δc^) limb buds (from E9.75: 27 somites to E10.5: 35 somites) points to a failure in establishing posterior identity upstream of *Shh* activation. Open arrowheads: loss of expression in *Hand2* deficient forelimb buds; solid arrowheads: normal expression in wild-type and *Shh* deficient limb buds. By E10.25–10.5 the posterior expression of *Tbx2* and *Tbx3* is also down-regulated in *Shh* ^Δ/Δ^ limb buds. (C–E) Posterior expansion of anterior markers in *H2* ^Δ/Δc^ limb buds. (C) *Crystallin-μ* (*Cry-μ*) is expressed ectopically in the posterior mesenchyme of *H2* ^Δ/Δc^ limb buds at E10.0 (30 somites; indicated by solid arrowheads). The ectopic posterior *Cry-μ* expression is detected earlier than in *Hand2* than *Shh* deficient limb buds (not shown). The *Alx4* (D) and *Gli3* (E) expression domains are posteriorly expanded (indicated by arrows) in *Hand2* deficient limb buds at E9.75 (27 somites) and E10.25 (32 somites), respectively. Note that the posterior expansion of the *Gli3* expression domain is less pronounced in *Shh* ^Δ/Δc^ than in *H2* ^Δ/Δc^ limb buds. In all panels, limb buds are oriented with the anterior to the top and the posterior to the bottom.

Our further analysis focused on the most severe, complete *Hand2* loss-of-function phenotypes in forelimb buds (Figure 1). The early essential requirement of Hand2 upstream of SHH in forelimb buds (for cell survival, Figure 1D) is further substantiated by molecular analysis, which reveals the lack of *Tbx3* and *Tbx2* expression [37] in the posterior mesenchyme of *H2* ^Δ ˜Δc^ forelimb buds. In contrast, their posterior expression is initiated but not up-regulated in *Shh* ^Δ ˜Δ^ forelimb buds (Figure 3A and 3B). The expression of *5′HoxD* genes is activated but not propagated in *Hand2* deficient limb buds (Figure S4A, S4B), likely due to the disruption of SHH signaling (Figure 1C). Concurrently, the expression of anterior genes such as *Cry-μ*, *Alx4* and *Gli3* is ectopically activated or expands to the posterior margin in *H2* ^Δ ˜Δc^ forelimb buds earlier and/or more prominently than in *Shh* ^Δ ˜Δ^ limb buds (Figure 3C–3E and Figure S4C). This loss of posterior and gain of anterior molecular markers reveal the early essential requirement of Hand2 for establishing posterior limb bud identity.

### In wild-type limb buds, Hand2-containing chromatin complexes are bound to the ZRS *cis*-regulatory region that controls *Shh* expression

**Figure 4:**
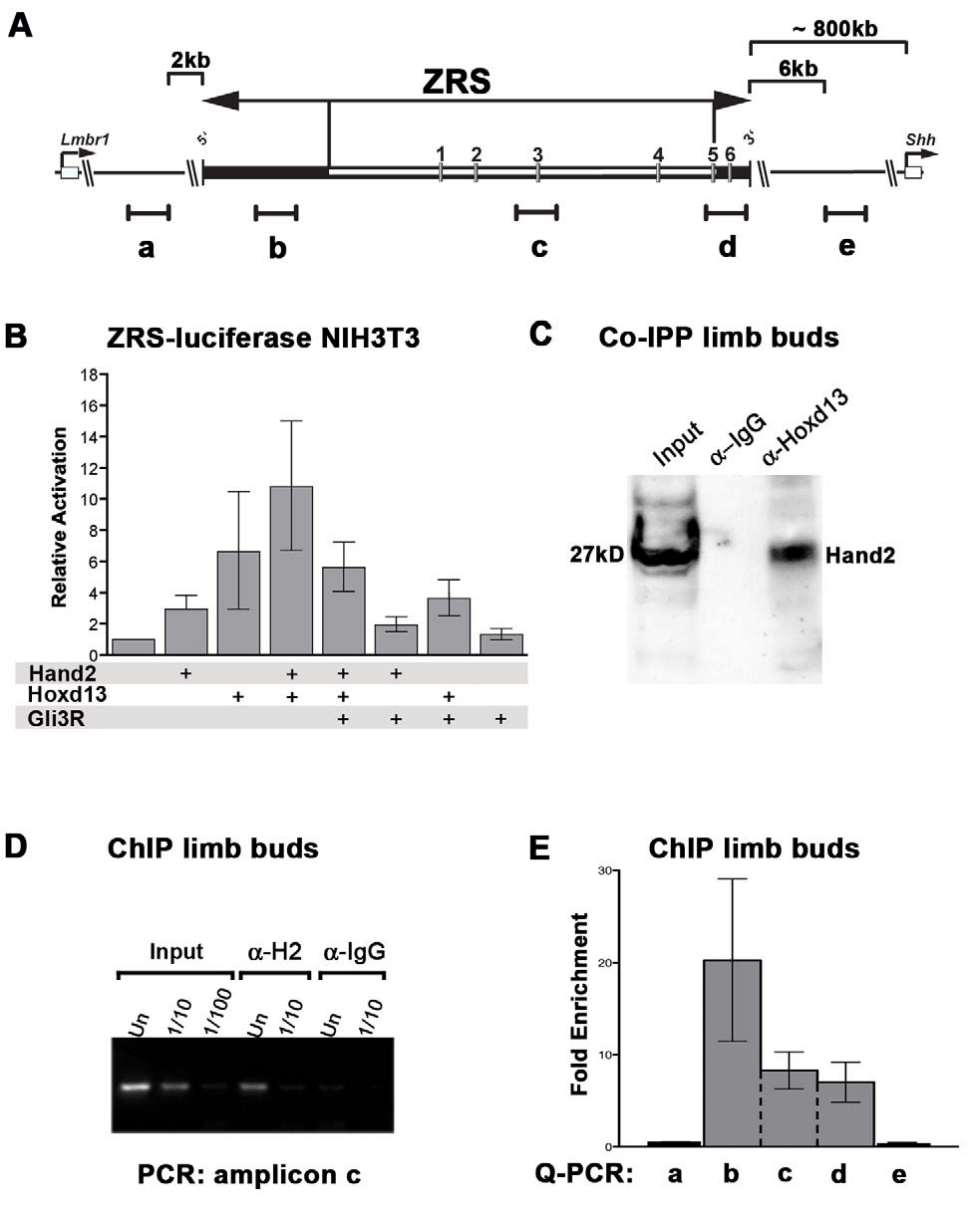
Hand2 interacts with Hoxd13 and is part of the chromatin complexes bound to the *ZRS* in limb buds. (A) Scheme of the ∼1.7 kb mouse ZRS *cis*-regulatory and the flanking genomic regions. The ZRS is located within an intron of the mouse *Lmbr1* gene (indicated on the left) and located ∼800 kb upstream of the *Shh* proximal promoter and coding exons (indicated on the right, see also Figure S5). The evolutionary conserved ZRS region drives expression of a *LacZ* reporter gene in *Shh*-like pattern in mouse limb buds [28], while deletion of the MFCS1 core region (indicated in white) disrupts *Shh* activation in limb buds [29]. Six *Ebox* sequences in the ZRS, which could potentially interact with Hand2 proteins are numbered “1” to “6”. Black lines indicate the approximate positions and sizes of the PCR amplicons for ChIP analysis. Note that amplicons “b” to “d” reside within the mouse ZRS, while amplicons “a” and “e” are located ∼2 kb upstream and ∼6 kb downstream of the ZRS and serve as non–ZRS controls. (B) Luciferase transactivation assay in NIH3T3 fibroblasts. Cells were co-transfected with ZRS-Luc and the expression plasmids indicated. Bars represent standard deviations. P<0.0001 for all samples except Gli3R alone: P = 0,0519. (C) Co-immunoprecipitation of Hand2 and Hoxd13 from wild-type limb buds (E10.5) using anti-Hoxd13 antibodies (α-Hoxd13) or IgGs (control). Hand2 proteins associated to Hoxd13 protein complexes were detected by Western blotting. (D,E) ChIP from wild-type limb buds (E11.0) to detect Hand2-containing chromatin complexes bound to the ZRS. (D) Analysis of amplicon “c” by conventional PCR (186 bp). Input: DNA isolated from cross-linked chromatin of E11.0 limb buds prior to ChIP was used as a positive control for PCR amplification. α-H2: ChIP using Hand2 antibodies. α-IgG: ChIP using non-specific goat IgGs as a control. Un: undiluted sample; dilutions as indicated. (E) Q–PCR analysis of three completely independent ChIP experiments using freshly cross-linked chromatin and α-Hand2 antibodies. The average values ± standard error are shown. Values obtained by amplifying a particular region from ChIP experiments using non-specific goat IgGs were arbitrarily set at 1 and used to calculate the values for the α-Hand2 ChIP experiments. Statistical evaluation by the Mann-Whitney test shows that the amplicons within the ZRS (“b” to “d”) are enriched in a statistically highly significant manner in comparison to the adjacent non-ZRS amplicons (“a” and “e”; p = 0.0018).

This analysis (Figure 1, Figure 2, Figure 3) led us to consider the possibility that Hand2 might directly transactivate *Shh* expression, possibly in conjunction with 5′Hox genes, which are essential for *Shh* activation in mouse limb buds [24],[26]. Chromatin immunoprecipitation (ChIP) studies showed previously that Hoxd13 containing chromatin complexes are bound to the far up-stream ZRS *cis*-regulatory region that controls *Shh* expression in limb buds [27]. In addition, Hoxd13 is able to transactivate a ZRS-luciferase reporter construct in transfected cells [27]. Therefore, the potential direct interactions of Hand2 with Hoxd13 proteins and the ZRS were assessed by luciferase transactivation assays in NIH3T3 cells, which are mouse fibroblasts commonly used to analyze the SHH pathway [38]. A luciferase reporter construct encoding the entire ZRS (ZRS-Luc) was generated by inserting the ∼1.7 kb mouse ZRS region (Figure 4A and Figure S5) [28] upstream of an adenovirus minimal promoter (for details see Text S1). The basal activity of this ZRS-Luc reporter construct was set to 1 and transfection of either Hand2 (∼3-fold) or Hoxd13 (∼6.5-fold) induced luciferase activity and their co-transfection resulted in an ∼10.5-fold increase (Figure 4B). *In silico* analysis revealed 6 *bona fide Ebox* sequence elements within the ZRS (Figure 4A and Figure S5). Inactivating point mutations in either individual or several of these *Ebox* elements reduce the activity of the ZRS, but not in a strictly Hand2-dependent manner as the transactivation by Hoxd13 alone is also affected (data not shown). As Hand2 and Gli3R act in a mutually antagonistic manner during initiation of limb bud development [11], the potential effects of Gli3R on transactivation were assessed. As neither the Gli3 nor Gli1 activator forms are able to activate the ZRS-Luc reporter on their own (data not shown), the ZRS likely lacks functional *Gli* binding sites [39], suggesting that any effects of Gli3R would be indirect. Indeed, co-expression of Gli3R results in significant inhibition of transactivation in the presence of Hoxd13 (Figure 4B), in agreement with the proposal that Gli3R can bind to and potentially antagonize Hoxd13 function [33]. In particular, Gli3R represses Hand2-Hoxd13 mediated transactivation of the ZRS-Luc reporter by ∼50% (Figure 4B).

The relevance of these interactions for limb bud development was determined by co-immunoprecipitation (Figure 4C and Figure S6) and ChIP analysis (Figure 4D and 4E). Immunoprecipitation of Hoxd13 proteins in combination with Western blotting reveals the existence of protein complexes containing both Hoxd13 and Hand2 protein in wild-type limb buds (Figure 4C). The likely direct nature of these interactions is supported by efficient co-precipitation of epitope-tagged Hand2 and Hoxd13 proteins from transfected cells (Figure S6). These experiments establish that Hand2 interacts directly with Hoxd13 but not with Gli3R (Figure S6), which is relevant with respect to their genetic interaction (see below). As the available polyclonal Hand2 antibodies specifically recognize and immunoprecipitate Hand2 proteins (Figure S2B, S2C, S2D), ChIP on wild-type mouse limb buds was performed [40] to enrich Hand2 containing chromatin complexes and the analysis of three independent, fresh chromatin preparations is shown in Figure 4D and 4E. Conventional PCR using the amplicon “c” (Figure 4A) detected this ZRS region in chromatin precipitated with anti-Hand2 antibodies (lanes α-H2, Figure 4D), while no such amplification was detected when non-specific IgGs were used (lanes α-IgG; Figure 4D). To further analyze this apparent association of Hand2 containing chromatin complexes with the ZRS, three amplicons (“b”, “c”, “d”) probing different regions of the ∼1.7 kb mouse ZRS (Figure 4A) were used for real-time PCR (Q-PCR) analysis. In addition, two amplicons located outside the mouse ZRS were chosen as likely negative controls (non-ZRS amplicons “a” and “e” in Figure 4A and 4E and Figure S5). Indeed, Q-PCR analysis revealed a minimally 14-fold enrichment of the amplicons located within the ZRS in comparison to the adjacent non-ZRS regions (Figure 4E). This enrichment is specific as ChIP using non-specific IgGs resulted in much lower Q-PCR amplification of all five regions. In particular, the enrichment of the ZRS in comparison to flanking non-ZRS regions is highly significant (amplicons “b” to “d” versus “a” and “e”; p = 0.0018), while the variability among the three ZRS amplicons is not significantly different. Interestingly, the ZRS region encompassing amplicon “b”, whose enrichment is most variable, does not encode any *bona fide Ebox* elements (Figure 4A and 4E). This provides additional evidence for the fact that the interaction of Hand2-containing chromatin complexes with the ZRS may not depend only on *Ebox* sequences. This ChIP analysis (Figure 4D and 4E) provides good evidence that the Hand2-containing chromatin complexes bind to the ZRS *cis*-regulatory region, but not to adjacent non-ZRS sequences.

### Mouse limb buds deficient for both *Hand2* and *Gli3* lack AP asymmetry along the entire PD axis and are severely polydactylous

**Figure 5:**
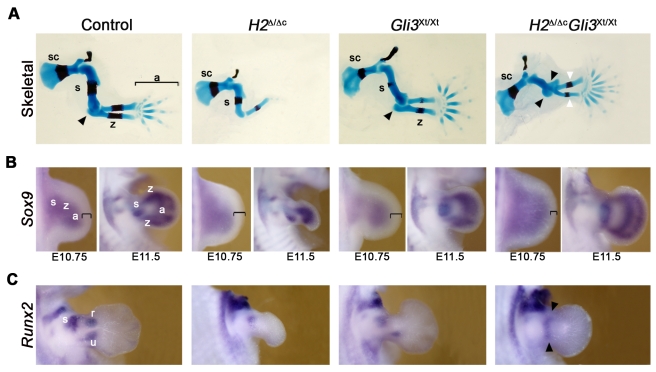
Forelimb buds lacking *Hand2* and *Gli3* lack AP polarity along the entire PD axis. (A) Skeletal preparations of *H2* ^Δ/Δc^ *Gli3* ^Xt/Xt^, *H2* ^Δ/Δc^, and *Gli3* ^Xt/Xt^ single mutant and control (*H2* ^Δ./^) forelimbs at E14.5. The black arrowheads point to the duplicated elbow-like structure while the white arrowheads point to the symmetrical zeugopodal skeletal elements in *H2* ^Δ/Δc^ *Gli3* ^Xt/Xt^ limbs. Note the shortening of the stylopod in double mutant limbs. (B) Expression of *Sox9* in limb buds at E10.75 (38 somites) and E11.5. Black brackets indicate the non-expressing distal mesenchyme that is reduced in *H2* ^Δ/Δc^ *Gli3* ^Xt/Xt^ limb buds. (C) *Runx2* expression in wild-type limb buds marks the presumptive stylopod (s) and zeugopodal domains (r/u) at E12.0. Note that establishment of anterior expression domain is delayed in *Gli3* ^Xt/Xt^ mutant limbs as it becomes visible by E12.5 (data not shown). Black arrowheads point to the apolar proximal expression of *Runx2* in *H2* ^Δ/Δc^ *Gli3* ^Xt/Xt^ mutant limb buds. In wild-type limb buds, the presumptive expression domains for *Sox9* and *Runx2* are indicated as previously defined [42],[63]. sc: scapula; s: stylopod; z: zeugopod; a: autopod; u: ulna; r: radius. All limb buds are oriented with the anterior to the top and the posterior to the bottom.

**Figure 6:**
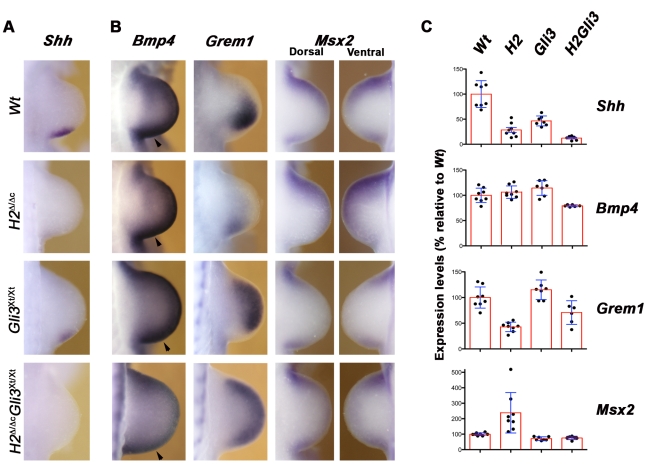
*Shh* expression and BMP pathway activity in *H2* ^Δ/Δc^ and *H2* ^Δ/Δc^ *Gli3* ^Xt/Xt^ forelimb buds. (A) No *Shh* expression is detected in the posterior mesenchyme of *H2* ^Δ/Δc^ and *H2* ^Δ/Δc^ *Gli3* ^Xt/Xt^ limb buds at E10.25 (32–33 somites). (B) *Bmp4, Grem1*, and *Msx2* expression at E10.5 (34–35 somites). Note that *Grem1* expression is activated, but not up-regulated and expanded distal-anterior in *H2* ^Δ/Δc^ limb buds. In contrast, the Grem1 expression domain appears rather uniform in the majority of all *H2* ^Δ/Δc^ *Gli3* ^Xt/Xt^ limb buds. (C) Q–PCR quantitation of *Shh*, *Bmp4*, *Grem1* and *Msx2* expression in single limb buds of mouse embryos of the indicated genotypes at ∼E10.5 (34–37 somites). Boxes show the average (± standard deviation), dots indicate levels in individual limb bud determined by triplicate analysis. The vertical axis indicates expression levels in percentages of wild-type levels (wild-type average set at 100%). Wt: wild-type (n = 8 single limb buds analyzed); *H2*: *H2* ^Δ/Δc^ (n = 8); *Gli3*: *Gli3* ^Xt/Xt^ (n = 7); *H2Gli3*: *H2* ^Δ/Δc^ *Gli3* ^Xt/Xt^ (n = 6). All differences discussed in the text are statistically highly significant (p-values between p<0.001 and p<0.05 using Mann-Whitney tests).

**Figure 7:**
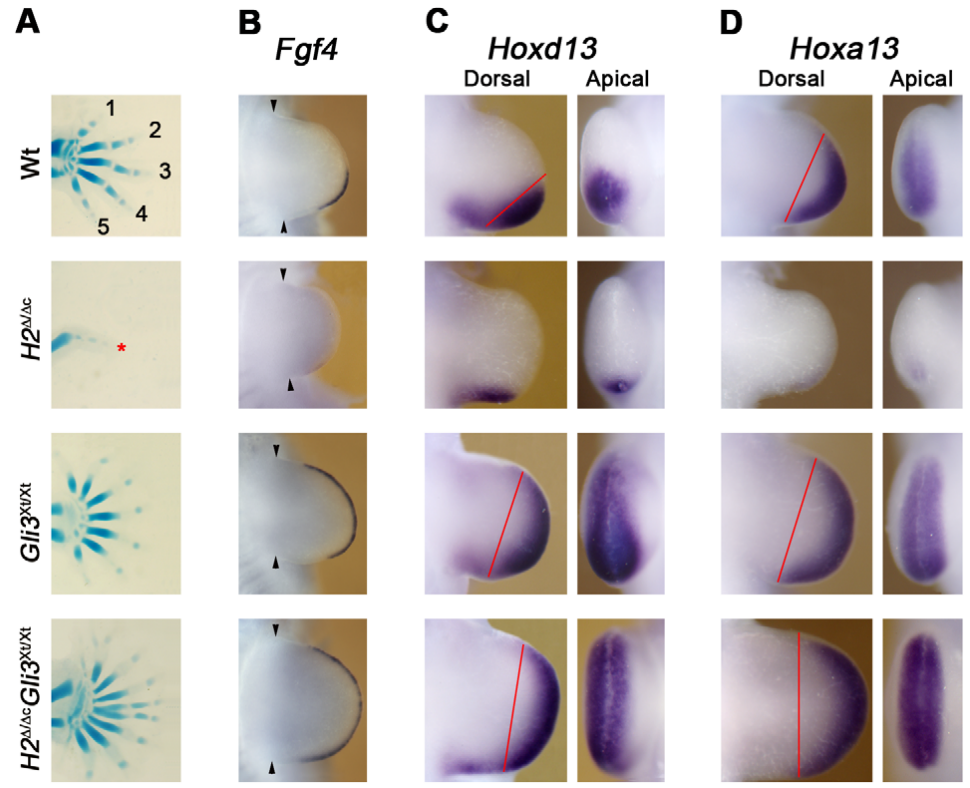
Apolar expression of *Fgf4*, *Hoxd13*, and *Hoxa13* in the autopod primordia of *H2* ^Δ/Δc^ *Gli3* ^Xt/Xt^ forelimb buds. (A) Skeletal preparations of the autopod (E14.5) of *H2* ^Δ/Δc^ *Gli3* ^Xt/Xt^, *H2* ^Δ/Δc^, and *Gli3* ^Xt/Xt^ single mutant and wild-type forelimbs. Digit identities are indicated by numbers 1 (thumb, anterior) to 5 (little finger, posterior). Black asterisks indicate digits with undetermined identities; red asterisk indicates the rudimentary digit formed in *H2* ^Δ/Δc^ forelimbs. (B) *Fgf4* expression in the AER of wild-type and mutant limb buds at E10.5 (36 somites). *Fgf4* is expressed at very low levels in the posterior of in *H2* ^Δ/Δc^ limb buds, but expands throughout the AER of *H2* ^Δ/Δc^ *Gli3* ^Xt/Xt^ forelimb buds. Arrowheads indicate the anterior and posterior margins of limb buds. (C) *Hoxd13* expression at E10.75 (40 somites). The late *Hoxd13* expression domain in *H2* ^Δ/Δc^ *Gli3* ^Xt/Xt^ limb buds appears symmetrical in contrast to e.g. *Gli3* deficient limb buds (expression borders are indicated by red lines). This is best seen by comparing apical views. (D) *Hoxa13* expression at E10.75 (41 somites). The *Hoxa13* expression domain appears also symmetrical in *H2* ^Δ/Δc^ *Gli3* ^Xt/Xt^ limb buds, while some asymmetry is retained in *Gli3* deficient limb buds (red lines in dorsal views; best seen by comparing the apical views).

As embryos lacking *Hand2* in limb buds survive to advanced stages (Figure 1B), the functional relevance of the pre-patterning mechanism [11] can now be genetically investigated in *Hand2* and *Gli3* compound mutant (*H2* ^Δ/Δc^ *Gli3* ^Xt/Xt^) embryos (Figure 5, Figure 6, Figure 7). In contrast to the *Hand2* deficiency, *H2* ^Δ/Δc^ *Gli3* ^Xt/Xt^ limbs are severely polydactylous and display little phenotypic variability (Figure 5A and Figure S7A). In addition, the zeugopodal bones and elbow joints appear strikingly symmetrical (Figure 5A, white and black arrowheads in panel *H2* ^Δ ˜Δc^ *Gli3* ^Xt/Xt^). These limb skeletal abnormalities are much more severe than the ones of *Gli3* ^Xt/Xt^ and *Shh* ^Δ ˜Δ^ *Gli3* ^Xt/Xt^ limbs (Figure 4A, panel *Gli3* ^Xt/Xt^; see also [9],[10]). While the skeletal elements of *H2* ^Δ ˜Δc^ *Gli3* ^Xt/Xt^ limbs seem to lack AP asymmetry, survival of the zeugopod and autopod progenitors is restored and the primordia are expanded in contrast to *H2* ^Δ ˜Δc^ limbs (Figure S7B and data not shown). Moreover, the *Sox9* expression domain, which marks the pre-chondrogenic lineage [41], is expanded in *H2* ^Δ ˜Δc^ *Gli3* ^Xt/Xt^ limb buds that tend to be larger than normal (Figure 5B, panel *H2* ^Δ ˜Δc^ *Gli3* ^Xt/Xt^). However, no significant changes in proliferation were observed in *H2* ^Δ ˜Δc^ *Gli3* ^Xt/Xt^ limb buds (data not shown). While the pre-chondrogenic condensations of all major skeletal elements are discernible by E10.75 in wild-type and *Gli3* deficient limb buds, *Sox9* expression remains diffuse and non-polarized in *H2* ^Δ ˜Δc^ *Gli3* ^Xt/Xt^ limb buds (Figure 5B). During autopod development, the pool of *Sox9* expressing digit progenitors is significantly expanded in *H2* ^Δ ˜Δc^ *Gli3* ^Xt/Xt^ limb buds in comparison to *Gli3* mutants and wild-types (Figure 5B; compare limb buds at E11.5). The apparent symmetry of in particular the zeugopod in the *H2* ^Δ ˜Δc^ *Gli3* ^Xt/Xt^ limbs contrasts with the normal AP asymmetry in *Gli3* ^Xt/Xt^ and *Shh* ^Δ ˜Δ^ *Gli3* ^Xt/Xt^ limbs (Figure 5A) [9]. This observation indicates that *Hand2* and *Gli3* participate in establishment of the AP asymmetry of the proximal limb skeleton independent of SHH signaling. Indeed, the expression of *Runx2*, which marks proximal skeletal primordia [42], is altered in double mutant limb buds (Figure 5C). By E12.0, *Runx2* is expressed in the presumptive stylopod and zeugopodal domains of wild-type limb buds, while few *Runx2* positive cells are detected in *Hand2* deficient limb buds (Figure 5C). In contrast, the *Runx2* expression domain is expanded and lacks polarity in the proximal part of double mutant limb buds (Figure 5C, black arrowheads). Taken together, these results indicate that the skeletal phenotypes and the severe polydactyly of *H2* ^Δ ˜Δc^ *Gli3* ^Xt/Xt^ limbs arise as a consequence of disrupting AP asymmetry (proximally as indicated by *Runx2*) and aberrant expansion of the skeletal progenitor pools (distally as indicated by *Sox9*).

### Disruption of the self-regulatory system that interlinks the SHH, BMP, and FGF signaling pathways in limb buds

In *H2* ^Δ ˜Δc^ *Gli3* ^Xt/Xt^ limb buds, *Shh* expression is not detected by *in situ* hybridization (Figure 6A) and its expression is ∼10-fold lower than in wild-types (Figure 6C). Interestingly, the variability in *Shh* expression following *Prx1-*Cre mediated inactivation of *Hand2* (Figure 1C, Figure S3B, S3C, S3D, and Figure 6C) is no longer observed in *H2* ^Δ ˜Δc^ *Gli3* ^Xt/Xt^ limb buds (Figure 6A and 6C), which agrees with the lack of significant variability in the resulting skeletal phenotypes (Figure 5A). This could be linked to the fact that posterior Shh expression is already reduced by ∼50% in *Gli3* ^Xt/Xt^ limb buds (Figure 6A and 6C). The low *Shh* transcript levels detected in the most severely affected *H2* ^Δ ˜Δc^ and *H2* ^Δ ˜Δc^ *Gli3* ^Xt/Xt^ limb buds (between 8% and 20%, Figure 6C) likely reflect basal expression not detected by *in situ* hybridization (Figure 1D, Figure 6A; see Discussion). BMP4-mediated up-regulation of its antagonist *Grem1* in the posterior mesenchyme is essential to initiate the self-regulatory signaling system that promotes distal limb bud development [43],[44]. In *H2* ^Δ ˜Δc^ limb buds, *Bmp4* expression appears not significantly altered, while its expression is slightly reduced in *H2* ^Δ ˜Δc^ *Gli3* ^Xt/Xt^ limb buds (panels *Bmp4* in Figure 6B and 6C). In particular, the posterior expression domain in double mutant limb buds appears smaller (arrowheads, panels *Bmp4* in Figure 6B), which results in rather symmetrical *Bmp4* expression along the AP limb bud axis. Furthermore, *Grem1* expression is activated, but not up-regulated and distal-anteriorly expanded in *Hand2* deficient limb buds (panel *Grem1* in Figure 6B), similar to *Shh* deficient limb buds [44]. In double mutant limb buds, the *Grem1* expression domain appears symmetrical due to its anterior expansion. However, the rather variable *Grem1* transcript levels are overall reduced in *H2* ^Δ ˜Δc^ *Gli3* ^Xt/Xt^ limb buds in comparison to wild-type and *Gli3* deficient limb buds (panels *Grem1* in Figure 6C). Finally, the expression of the direct BMP transcriptional target *Msx2* [43] is expanded in *H2* ^Δ ˜Δc^ limb buds, while its expression is significantly reduced in *Gli3* deficient and double mutant limb buds as a likely consequence of the alterations in *Grem1* (panels *Msx2* in Figure 6B and 6C). Taken together, these results corroborate the proposal that the initial phase of *Grem1* expression in the posterior mesenchyme depends on BMP4 activity [43]. The rather symmetrical *Grem1* expression in *H2* ^Δ ˜Δc^ *Gli3* ^Xt/Xt^ limb buds indicates that the second phase of SHH-dependent distal-anterior expansion of its expression in wild-type limb buds is a likely consequence of SHH-mediated inhibition of Gli3R activity [6].

### Loss of AP asymmetry in the autopod of *H2* ^Δ ˜Δc^ *Gli3* ^Xt/Xt^ limb buds

The lack of discernible AP identities in the autopod of *H2* ^Δ ˜Δc^ *Gli3* ^Xt/Xt^ limb buds (Figure 7A) is confirmed by molecular analysis. In agreement with the rather symmetric distribution of *Bmp4* and *Grem1* in the distal limb bud mesenchyme (Figure 6B), *Fgf4* is expressed uniformly by the AER in double mutant limb buds (Figure 7B). The distal expression domains of the *Hoxd13* and *Hoxa13* genes mark the presumptive autopod territory and are required for specification and expansion of the digit progenitors [45],[46]. Within the distal mesenchyme of *H2* ^Δ ˜Δc^ *Gli3* ^Xt/Xt^ forelimb buds, the expression of *Hoxd13* is anteriorly expanded and appears apolar in comparison to wild-type and *Gli3* mutant limb buds (Figure 7C; best seen in the apical views). In addition, the AP asymmetry of the distal *Hoxa13* domain is also lost in double mutant limb buds (Figure 7D; best seen in the apical views). The expanded and apolar expression of these genes (Figure 7B–7D) together with the alterations in *Sox9*, *Runx2* (Figure 5B and 5C), *Bmp4* and *Grem1* (Figure 6B) reveal the striking loss of the asymmetrical expression of molecular and cellular markers of the AP axis along the entire PD axis in limb buds lacking both *Hand2* and *Gli3*.

## Discussion

In this study, we uncover the key regulatory interactions involving Hand2 that control establishment of posterior limb bud identity upstream of SHH signaling, in particular the genetic interactions with Gli3 that initiate AP axis polarity. Secondly, we reveal that Hand2, which like 5′Hox genes is essential for establishment of the *Shh* expressing limb bud organizer in the posterior-proximal mesenchyme, is part of the chromatin complexes bound to ZRS *cis*-regulatory region. The striking loss of posterior and gain of anterior molecular markers in *Hand2* deficient limb buds indicates that limb field symmetry may normally be broken by Gli3R-mediated posterior restriction of *Hand2* expression. This most likely parallels activation of *5′HoxD* genes in the posterior mesenchyme [45]. In *Hand2* deficient limb buds, the SHH dependent establishment of the late *5′HoxD* expression domains is disrupted, while in limb buds lacking both *Hand2* and *Gli3*, the late *5′HoxD* expression domains expand uniformly throughout the distal autopod. Therefore, the down-regulation of *5′HoxD* genes in *Hand2* deficient limb buds is a likely consequence of increased Gli3R activity due to lack of SHH signaling [23]. Furthermore, *Hand2* participates in transcriptional activation and/or upregulation of *Tbx2/3* and *Shh* expression in the posterior mesenchyme and is required for anterior restriction of *Gli3* and *Alx4* expression. In *Hand2* deficient limb buds, expression of the BMP antagonist *Grem1* is activated in the posterior mesenchyme under the influence of BMP signaling (ref. 43 and this study). This previous analysis and the observed anterior expansion of *Grem1* expression in *H2* ^Δ ˜Δc^ *Gli3* ^Xt/Xt^ limb buds reveals that the transcriptional activation and positioning of the *Grem1* expression domain is controlled by interaction of BMP4 (positive) with GLI3R (negative). In wild-type limb buds, the *Grem1* expression domain is always located distal-anterior to the *Shh* expressing cells and their descendents [47],[48], while it remains proximal and low due to the lack of SHH signaling in *H2* ^Δ ˜Δ^ limb buds (this study). Taken together, these results provide further insights into the molecular mechanism controlling spatial and temporal aspects of BMP4-mediated initiation and SHH-dependent progression of *Grem1* expression, which acts as an essential node in the self-regulatory signaling system that controls limb development [1].

### Hand2, the ZRS, and establishment of the *Shh* expression domain in the posterior limb bud mesenchyme

Our biochemical analysis of chromatin isolated from wild-type mouse limb buds reveals that Hand2-containing chromatin complexes are bound to the ZRS, which is the far upstream *cis*-regulatory region required for *Shh* expression in limb buds [28],[29]. In particular, ZRS sequences are specifically and significantly enriched in Hand2 containing chromatin complexes in contrast to flanking regions. Furthermore, Hand2 is part of Hoxd13 protein complexes in limb buds and in transfected cells, the two proteins transactivate the expression of a luciferase reporter gene in a ZRS-dependent manner. Albeit the fact that such transactivation studies are of somewhat artificial nature, the conclusions reached by this analysis completely agree with the results of our genetic analysis of *Hand2* functions during mouse limb bud development. Early and complete genetic inactivation of Hand2 in limb buds disrupts establishment of the *Shh* expression domain in the posterior limb bud, while either incomplete or temporally delayed inactivation does no longer disrupt initiation of *Shh* expression (this study). This reveals the early essential requirement of Hand2 for establishment of the posterior *Shh* expression domain, while subsequently Hand2 appears to contribute to transcriptional up-regulation of *Shh* expression. This may happen as part of an auto-regulatory loop because SHH signaling in turn up-regulates *Hand2* expression most likely via repressing production of the Gli3R isoform [9],[11],[49]. The low levels of *Shh* expression detected by Q-PCR even in the most affected *H2* ^Δ ˜Δc^ and *H2* ^Δ ˜Δc^ *Gli3* ^Xt/Xt^ limb buds, but not in *Shh* deficient limb buds (JDB and RZ, unpublished) are indicative of basal transcription of the *Shh* locus in the absence of *Hand2*, which is not detectable by *in situ* hybridization (this study). This basal expression may depend on Hox transcription factors [24],[26] or other regulators of *Shh* expression in limb buds (see below). However, our study shows that Hand2 is essential to establish and upregulate *Shh* expression in the posterior mesenchyme, which defines the SHH signaling limb bud organizer [1]. This Hand2-mediated transactivation of *Shh* expression is a likely consequence of its direct interaction with the ZRS *cis*-regulatory region and is possibly enhanced by formation of transcriptional complexes with Hoxd13 protein in limb buds.

Genetic and experimental manipulation of paired appendage buds in mouse, chicken and zebrafish embryos have begun to reveal the factors required in addition to *Hand2* and *5′HoxD* genes for *Shh* activation. In particular, AER-FGF and retinoic acid signaling have also been implicated in the activation of *Shh* expression [21],[50]. Deletion of both the *HoxA* and *HoxD* clusters in mouse embryos disrupts *Shh* activation and causes early arrest of limb bud development such that the limb skeleton is truncated at the level of the stylopod [24],[26]. But in contrast to *Hand2*, loss-of-function mutations in these genes alone or in combination do not phenocopy the *Shh* loss-of-function limb skeletal phenotypes [51],[52]. The Hand2 protein interacts with Hoxd13 and is part of the chromatin complexes bound to the ZRS in limb buds (this study). However, other transacting factors will likely contribute to ZRS dependent activation of *Shh* transcription. In fact, the overlap of the *Hand2* and *Hoxd13* expression domains in the posterior limb bud mesenchyme is much bigger than the initial *Shh* expression domain. During limb bud initiation stages, the *Hand2* and *Gli3* expression domains overlap significantly, but then become rapidly mutually exclusive [11]. Therefore, these early dynamic changes in the expression domains of the *Hand2*, *Gli3* and *Hoxd13* transcriptional regulators may well alter their interactions and spatially restrict the formation of transcription initiating/enhancing Hand2-Hoxd13 chromatin complexes at the ZRS to the posterior limb bud (this study). These direct interactions would restrict the up-regulation of *Shh* expression to the posterior limb bud mesenchyme, thereby establishing the SHH signaling limb bud organizer. A recent study shows that the distant ZRS is in close proximity to the *Shh* transcription unit in both the anterior and posterior limb bud mesenchyme, but only loops out of its chromosomal territory in the posterior mesenchyme [31]. Interestingly, *Shh* is apparently transcribed by only a fraction of all ZPA cells at one particular time point, which indicates that the chromosomal conformation dynamics control *Shh* expression at the cellular level [31].

It is known that Hand2 binds DNA primarily as a heterodimer with E12 and/or the bHLH transcription factor Twist1 [16],[19]. Interestingly, *Twist1* is also required during early limb bud development [18] and point mutations in the human *Twist1* gene alter its dimerization with Hand2, which causes congenital limb malformations [19]. Therefore, these additional factors may also participate in regulation of *Shh* expression. The expression of *Hand2* and *5′HoxD* genes is activated in parallel, but then they converge functionally on the ZRS to establish the *Shh* expression domain in the posterior limb bud (this study and ref. 24). Furthermore, the establishment of the posterior *Tbx2* and *Tbx3* expression domains is disrupted in *Hand2* deficient limb buds. The *cis*-regulatory elements controlling their expression are currently unknown, but it has been shown that *Tbx2* expression requires the overlying non-AER ectoderm [53]. Additional experimental and genetic evidence indicates that Tbx2 and Tbx3 act likely upstream of *Shh* to restrict its transcriptional activation to the posterior limb bud margin [53],[54]. In particular, ectopic expression of *Tbx3* in early chicken limb buds induces an anterior shift of the entire limb bud together with transient anterior expansion of *Hand2* expression [55]. These studies indicate that *Tbx* genes are part of the molecular circuits that position the limb bud, specify posterior identity and restrict activation of *Shh* to its posterior margin.

### Breaking limb bud symmetry

The genetic inactivation of the pre-patterning mechanism in *H2* ^Δ ˜Δc^ *Gli3* ^Xt/Xt^ limb buds disrupts establishment of AP asymmetry and self-regulatory limb bud signaling [43], while PD axis outgrowth and formation of all three major limb skeletal segments are the likely consequence of uniform AER-FGF signaling [2]. This results in a shortened and symmetric stylopod, zeugopod and a polydactylous autopod with highly dysmorphic digits. Similar to *H2* ^Δ ˜Δc^ *Gli3* ^Xt/Xt^ limb buds, limbs lacking *5′HoxD* genes and *Gli3* are also severely polydactylous but retain some polarity [56],[57]. Therefore, the loss of AP polarity along the entire proximo-distal axis is more severe than the phenotypes observed in limb buds lacking *Gli3* alone or in combination with genes such as *Shh*, *Alx4* or *5′HoxD* genes [9], [56]–[58]. Over-expression of *Hand2* in the entire limb bud mesenchyme results in a duplication of the anterior zeugopod (ulna) and posterior autopod (digits) [12], which indicates that disturbing the balance between Hand2 and Gli3 either by gene inactivation or over-expression alters AP polarity. Therefore, the balance of the opposing activities of Hand2 and Gli3R in concert with 5′HoxD genes may control specification of the AP limb axis independent and up-stream of SHH signaling. In mouse limb buds lacking the *Plzf* zinc finger protein, *5′HoxD* genes are uniformly expressed from early stages onwards and AP polarity is partially lost in combination hindlimb digit polydactyly [59].

It remains unclear why the digit polydactyly in *H2* ^Δ ˜Δc^ *Gli3* ^Xt/Xt^ forelimbs is more severe than the one of *Gli3* ^Xt/Xt^ (and *Shh* ^Δ ˜Δ^ *Gli3* ^Xt/Xt^ [9]) forelimbs. However, in *H2* ^Δ ˜Δc^ *Gli3* ^Xt/Xt^ forelimb buds, the distal expression domains of *Hoxa13* and *Hoxd13*, which delineate the autopod territory and function in digit development (see [refs. 24],[26] for further detail) are anteriorly expanded in comparison to *Gli3* deficient limb buds. Such anterior expansion may point to an enlarged pool of autopod/digit progenitors, which could underlie the more severe digit polydactyly. As discussed before, this expansion of the *Hoxa/d13* expression domains and the presumptive autopod territory are a likely consequence of the early loss of AP polarity along the entire PD axis in double mutant forelimb buds in contrast to *Gli3* ^Xt/Xt^ mutants. In particular, the *H2* ^Δ ˜Δc^ *Gli3* ^Xt/Xt^ forelimb skeletons bear some resemblance to the primitive paired appendages of Devonian fish and the polydactylous limbs of early tetrapods [60]. We shows that these rather “primitive” limb structures develop in the absence of pre-patterning (Hand2, Gli3) and the self-regulatory signaling system that interlinks the SHH, BMP and FGF signaling pathways, which are both key to normal limb skeletal development [1]. During tetrapod evolution, the symmetry of primitive polydactylous autopods from the Devonian period [61] was likely broken by beginning to set-up the regulatory interactions described in this study as they initiate posterior polarity up-stream or in parallel to their requirement for establishment of the SHH signaling limb bud organizer. The establishment of these transcriptional regulatory network acting upstream of SHH signaling might have enabled the development of the more refined and better functional pentadactylous limbs of modern tetrapods.

## Materials and Methods

All animal experiments were performed in accordance with Swiss law and have been approved by the regional veterinary and ethics authorities.

### Mice and embryos

The generation of *Hand2* conditional mutant mice is shown in Figure S1. *Hand2* mouse strains were kept in a mixed 129SvJ/C57BL6 genetic background. For details of the generation and analysis of *Hand2* mice and embryos see Text S1.

### Immunoprecipitation (IP) and co-IP experiments

For IP, fore- and hind-limb buds from E11.0 embryos were collected in PBS and lysed in lysis buffer (Tris-HCl 10 mM pH 8.0; EDTA 1 mM; NaCl 140 mM; Triton 1%; SDS 0.1%; NaDeoxycholate 0.1%). Protein lysates (about 300 mg) were incubated overnight at 4°C with the anti-Hand2 (M-19, Santa Cruz; 1 mg) and protein G beads were added the next morning for about 5 hours at 4°C. After several washes in lysis buffer, beads were resuspended in Laemmli loading buffer and SDS-PAGE was performed under non-reducing conditions. Goat IgG antibodies were used as control. For Co-IP of endogenous embryonic proteins, 50 limb buds at E10.5 were dissected in PBS and processed as described [33]. The Hoxd13 or control rabbit IgG antibodies used for co-IPs were covalently cross-linked to G protein beads and bound proteins were detected with Hand2 antibodies (AF3876, R&D System).

### Chromatin Immunoprecipitation (ChIP)

ChIP was performed using wild-type fore- and hindlimb buds at E11.0 (38–42 somites). For each experiment, 85 limb buds were dissected, pooled and the freshly cross-linked chromatin divided among the starting samples. The average size of the DNA fragments in the cross-linked and sonicated chromatin was ∼500–2000 bp. Samples were processed as described [62] with the following modifications: protein G magnetic beads (Dynabeads, Invitrogen) were pre-absorbed with goat IgG (1–2 mg for 30 ml of beads for each sample) for minimally 1 hour at 4°C. After washing them with BSA-PBS (5 mg/ml), the beads were added to the chromatin extracts and gently rocked for 1 hour at 4°C. Afterwards, beads were spun down and the chromatin in the supernatant transferred to a new tube and incubated overnight with Hand2 antibodies (M-19, Santa Cruz; 1 mg) or goat IgG antibodies as control (1 mg). The following day, 25 ml of beads were added and the DNA-immunocomplexes were precipitated for 4 hours at 4°C. ChIP-enriched DNA samples were amplified by Q-PCR and conventional PCR. To compute the enrichment for a particular amplicon, its values were compared with the ones of a completely unrelated amplicon within the mouse *β-actin* gene that provides an additional negative control. The *β-actin* gene is located ∼114 Mb downstream of the ZRS on mouse chromosome 5. The fold of enrichment was then calculated as the fold of increase in the specific signal in relation to the values obtained when using non-specific goat IgGs for ChIP (values set arbitrarily at 1). All oligos used are listed in Table S1. Three ChIP experiments were performed using completely independent and fresh (i.e. non-frozen) chromatin preparations. The values obtained were analyzed and the graphs shown in Figure 4D (means ± standard error) were drawn using the Prism Graphpad Software (La Jolla, USA). The statistical significance of all results was assessed using the Mann-Whitney test as part of the Prism software package.

### Luciferase assays

Mouse NIH3T3 fibroblasts were plated on 24-well plates and transfected using Lipofectamine LTX (Invitrogen) including a total of 500 ng of DNA. Reporter constructs were co-transfected with 100 ng of *Hand2* and/or *Hoxd13* and/or *Gli3* expression constructs in combination with a *Renilla* luciferase vector. A detailed description of the generation of the expression constructs is available in Text S1. Cells were collected 28–30 hours post-transfection and luciferase reporter assays were performed using the Dual Luciferase Kit (Promega). Each assay was repeated at least 10 times. It is important to note that NIH3T3 cells do not express the endogenous *Hand2*, *Hoxd13* and *Gli3* genes (data not shown). For the co-immuno-precipitation assays in cells see Text S1.

## Supporting Information

Figure S1 Generation and validation of the *Hand2* conditional allele. (A) Scheme depicting the *Hand2* gene targeting strategy. A targeting vector was constructed in order to flank both *Hand2* coding exons with *loxP* sites (blue triangles). An *EcoRV (ERV)* restriction site was inserted to enable screening of ES-clones by Southern blot analysis. The *PGK*-Neo-pA cassette was inserted into the construct 3′ to the *loxP* site for positive selection. This selection cassette is flanked with two *FRT* sites (green triangles) to enable excision by the flipase (FLPe) recombinase. For genomic Southern blot analysis, the 5′ probe (violet box) and the 3′ probe (orange box) were used. The PCR oligos and sizes of amplified bands are indicated. Arrows indicate the direction of transcription. To induce FRT and *loxP* mediated recombination at the *Hand2* locus, mice carrying the *Hand2* floxed-neo allele (*H2* ^fneo^) were intercrossed with FLPe and with *CMV*-Cre transgenic mice. (B) Southern blot analysis showing wild-type, the correctly recombined 4D7 ES-cell clone and DNA biopsies from mice heterozygous for the *H2* ^fneo^ and the *Hand2* floxed (*H2* ^f^) allele. The 5′ probe detects a 15 kb *ERV* fragment for the wild-type (Wt) locus, while an 8 kb *ERV* fragment is detected when the locus is correctly recombined. The 3′ probe detects a 7.3 kb wild-type *PacI* fragment and a 9.3 kb fragment in the correctly targeted allele. Following excision of the *PGK*-Neo-pA cassette, the 9.3 kb is reduced to a 7.5 kb fragment in the *H2* ^f^ allele. (C) PCR genotyping. (D) Morphology of mouse embryos at embryonic day E9.5–9.75 (25–27 somites). *Hand2* deficient embryos are growth retarded, the aortic and pericardial sac are dilated and branchial arches are malformed [13]. The heart (h), first (I) and second branchial arches (II) are indicated. Asterisks indicate the outgrowing forelimb buds. (E) LysoTracker Red (LysoT) analysis reveals the massive and generalized cell death in *Hand2* deficient embryos and limb buds at E9.5 (25 somites). a: anterior; d: dorsal; p: posterior; v: ventral.
(6.79 MB TIF)

Figure S2 Clearance of *Hand2* transcripts from mutant forelimb buds and specificity of α-Hand2 antibodies. (A) Q-PCR analysis to determine *Hand2* transcript levels in wild-type, *Hand2* floxed (*H2* ^f^), Hand2 heterozygous and *Hand2* deficient limb buds at E10.25–10.5 (33–35 somites; n = 6–8). Note that no *Hand2* transcripts are detected in *Hand2* deficient limb buds. Bars: ±standard deviation. asterisk: P = 0.0009. (B) Immunofluorescense using α-Hand2 antibodies (M-19, Santa Cruz) reveals the specific nuclear localization of Hand2 proteins in posterior (Wt-p) but not anterior (Wt-a) limb buds mesenchymal cells. No specific fluorescence is detected in mesenchymal cells isolated from *Hand2* deficient limb buds. (C) Hand2 proteins are cleared from *Hand2* deficient limb buds by embryonic day E10.5. Protein extracts were normalized for their vinculin content. (D) Immunoprecipitation (IPP) of Hand2 proteins from E11.0 limb buds. Hand2 proteins are detected by Western blotting. Control: α-IgG. Asterisks indicate the cross-reactivity with the light chains of the IgGs (control and α-Hand2) used for IPP.
(2.42 MB TIF)

Figure S3 Incomplete/delayed inactivation of *Hand2* in forelimb buds results in a hypomorphic phenotype. (A) Skeletal preparations of control (*Prx1-Cre* heterozygous) and *Hand2* deficient forelimbs at E14.5. Due to slight variability in *Prx1-Cre* mediated inactivation of the conditional *Hand2* allele in forelimb buds, three classes of skeletal phenotypes are observed. The most hypomorphic phenotype (Weak) results in formation of two misplaced zeugopodal bones, three anterior digits and a hypoplastic digit that resembles digit 4 (indicated by an asterisk). The arrowhead points to the twisted bones of the zeugopod. The less hypomorphic phenotype (Intermediate) results in formation of one zeugopodal bone and two digits. The null phenotype (Strong) is identical to the skeletal phenotypes observed in *Shh* deficient limb buds (Figure 1A). Asterisks indicate digits with unclear identities. (B) Analysis of *Hand2* expression reveals the variable nature of *Prx1-Cre* mediated inactivation of *Hand2* at E9.75 (28 somites). (C) This variability is also apparent when levels of SHH signal transduction are monitored by *Gli1* expression at E9.75 (27 somites). Complete absence of *Hand2* (B) and *Gli1* transcripts (C) was observed in 50% of all *Prx1*-Cre1, *Hand2* deficient limb buds (n = 4/8). The others display varying degrees of *Hand2* and *Gli1* expression. All limb buds are oriented with the anterior to the top and the posterior to the bottom. (D) Table summarizing the frequencies of the three classes of limb skeletal phenotypes observed in *Hand2* mutant forelimbs. This variability is in agreement with the fact, that developmentally slightly later *Hand2* inactivation in hindlimb buds results in almost normal *Shh* expression and limb skeletal development (Figure 2). Taken together, these results indicate that *Hand2* needs to be inactivated very early and rapidly during the onset of limb bud development to disrupt establishment of the posterior *Shh* expression domain.
(3.68 MB TIF)

Figure S4 Activation of *5′HoxD* genes and posterior expansion of *Gli3* expression in *Hand2* deficient limb buds. *Hoxd11* expression at E9.75 (27 somites) and E10.75 (36 somites). Expression of *Hoxd11* is initiated in limb buds lacking *Hand2* (arrowheads), but its up-regulation is disrupted. (B) *Hoxd13* expression is initiated, but rapidly down-regulated in *Hand2* deficient limb buds (arrowheads E10.5, 33 somites). (C) *Gli3* expression is expanded posteriorly in *Hand2* deficient limb buds at E10.0 (32 somites; compare white to black arrowhead). In *Shh* deficient limb buds, *Gli3* is not expanded to the posterior margin (compare white to open arrowheads). All limb buds are oriented with the anterior to the top and the posterior to the bottom. (D) Inactivation of *Hand2* alters Gli3 protein processing. Protein extracts prepared from limb buds of the indicated genotypes at E10.5 (35 somites) were analyzed by immunoblotting using α-Gli3 antibodies. The full-length Gli3 protein is about 190 kD, while the processed Gli3R isoform is about 83 kD. Note that Gli3R form is more abundant in *Hand2* and *Shh* deficient than in wild-type limb buds. Samples are normalized for their vinculin contents. The asterisk points to an unrelated cross-reacting protein.
(5.95 MB TIF)

Figure S5 The genomic landscape encompassing the mouse ZRS. Scheme depicting part of mouse chromosome 5 (Ensemble: *Mus musculus* genomic region from position 29621310 to 29662806) analyzed in the ChIP experiments by Q-PCR. The *Lmbr1* locus encodes the mouse ZRS (1.67 kb) within intron 4, which is about 800 kb away from the *Shh* locus. The 6 *Ebox* elements (1 to 6) located in the ZRS are indicated. The framed orange and blue boxes indicate the 20 kb downstream and upstream flanking regions. These two regions are shown in the enlargements and potential *Ebox* elements are indicated. Coding exons are represented by filled boxes. Amplicon a is located about 2 kb downstream and amplicon e about 6 kb upstream of the ZRS (the primers used for Q-PCR amplification are indicated by green arrows).
(0.32 MB PDF)

Figure S6 Evidence that Hand2 interacts directly with the Hoxd13 but not Gli3R protein. Co-immunoprecipitation reveals the direct interaction of Hand2 with Hoxd13 in HEK293T cells (Hand2: Flag-epitope tagged; Gli3R: Myc-epitope tagged). In contrast, Gli3R is unable to directly interact with Hand2, but binds to Hoxd13 [12]. Protein extracts were immunoprecipitated (IP) using the following antibodies: α-Flag for Hand2, α-Hoxd13 for Hoxd13, α-Myc for Gli3R and immunoblotted (IB) using the appropriate antibodies.
(2.52 MB TIF)

Figure S7 Morphological defects in limb buds lacking *Hand2* and *Gli3*. (A) The forelimb morphology of double mutant mouse embryos at E14.5. Note the stunted forelimbs and the extreme pre- and post-axial polydactyly in comparison to *Gli3* ^Xt/Xt^ limb buds. White brackets indicate forelimb length. Asterisks indicate digits with undetermined identities. (B) The massive apoptosis of mesenchymal cells in *Hand2* deficient limb buds is suppressed in limb buds lacking both *Hand2* and *Gli3*. Apoptotic cells were detected by TUNEL fluorescence on limb bud sections at E10.25 (33 somites). Sections are oriented with the anterior to the top and posterior to the bottom.
(3.99 MB TIF)

Table S1 Oligos used for the study. All primers used for genotyping of mice and embryos, Q-PCR analysis of *Hand2* transcripts, Q-PCR analysis of the ChIP experiments are listed. Conditions for use are available upon request.
(0.04 MB DOC)

Text S1 Supporting materials and methods.
(0.08 MB DOC)
