# Supplementary figures and images for: Distinct Roles of Hand2 in Initiating Polarity and Posterior Shh Expression during the Onset of Mouse Limb Bud Development
Source: PLoS Genet. 2010 Apr 8;6(4):e1000901. doi: 10.1371/journal.pgen.1000901 (PMC2851570; doi:10.1371/journal.pgen.1000901)

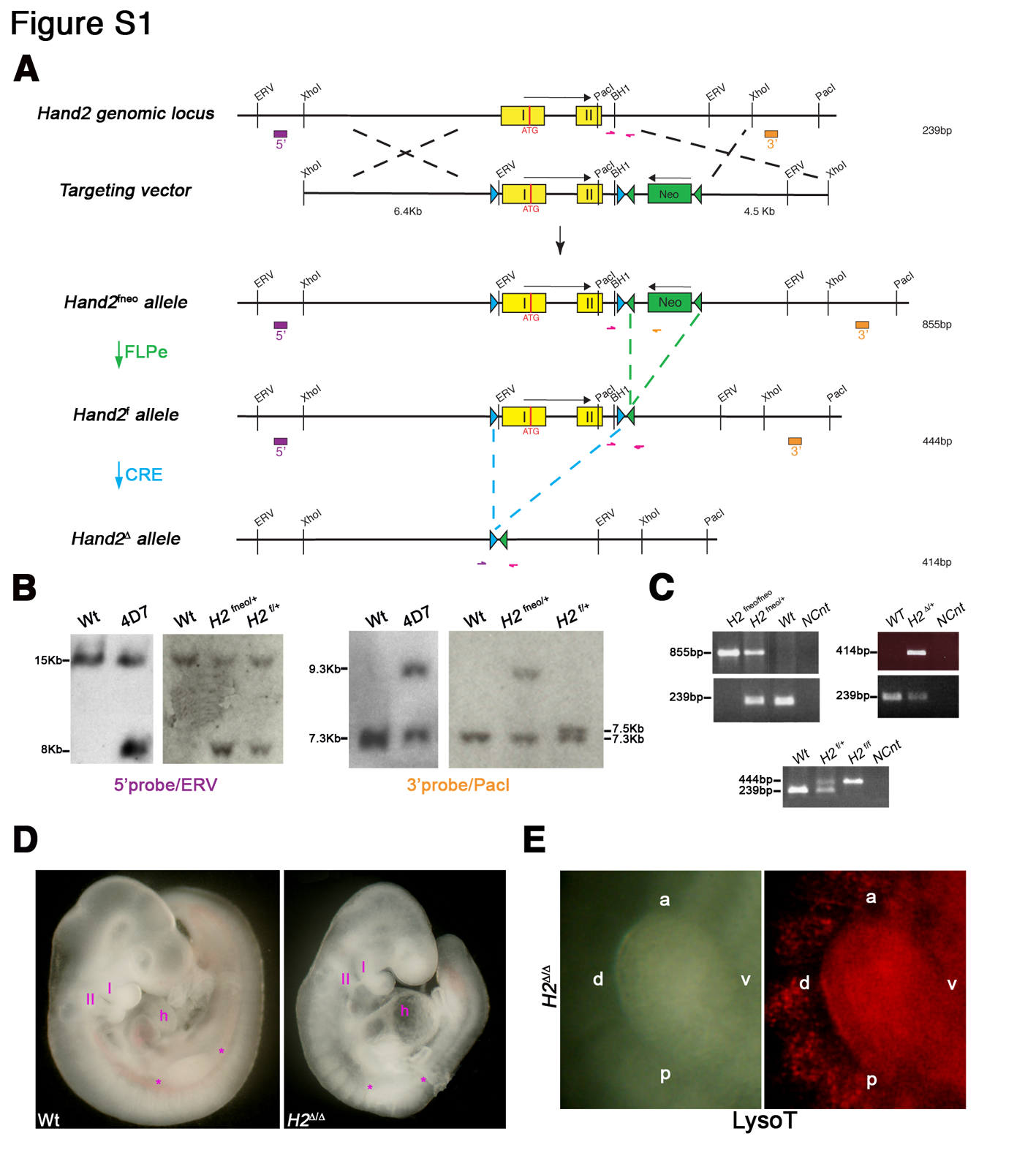

Supplement: Figure S1 — Generation and validation of the Hand2 conditional allele. (A) Scheme depicting the Hand2 gene targeting strategy. A targeting vector was constructed in order to flank both Hand2 coding exons with loxP sites (blue triangles). An EcoRV (ERV) restriction site was inserted to enable screening of ES-clones by Southern blot analysis. The PGK-Neo-pA cassette was inserted into the construct 3′ to the loxP site for positive selection. This selection cassette is flanked with two FRT sites (green triangles) to enable excision by the flipase (FLPe) recombinase. For genomic Southern blot analysis, the 5′ probe (violet box) and the 3′ probe (orange box) were used. The PCR oligos and sizes of amplified bands are indicated. Arrows indicate the direction of transcription. To induce FRT and loxP mediated recombination at the Hand2 locus, mice carrying the Hand2 floxed-neo allele (H2 fneo) were intercrossed with FLPe and with CMV-Cre transgenic mice. (B) Southern blot analysis showing wild-type, the correctly recombined 4D7 ES-cell clone and DNA biopsies from mice heterozygous for the H2 fneo and the Hand2 floxed (H2 f) allele. The 5′ probe detects a 15 kb ERV fragment for the wild-type (Wt) locus, while an 8 kb ERV fragment is detected when the locus is correctly recombined. The 3′ probe detects a 7.3 kb wild-type PacI fragment and a 9.3 kb fragment in the correctly targeted allele. Following excision of the PGK-Neo-pA cassette, the 9.3 kb is reduced to a 7.5 kb fragment in the H2 f allele. (C) PCR genotyping. (D) Morphology of mouse embryos at embryonic day E9.5–9.75 (25–27 somites). Hand2 deficient embryos are growth retarded, the aortic and pericardial sac are dilated and branchial arches are malformed [13]. The heart (h), first (I) and second branchial arches (II) are indicated. Asterisks indicate the outgrowing forelimb buds. (E) LysoTracker Red (LysoT) analysis reveals the massive and generalized cell death in Hand2 deficient embryos and limb buds at E9.5 (25 somites). a: ante [file pgen.1000901.s001.tif]

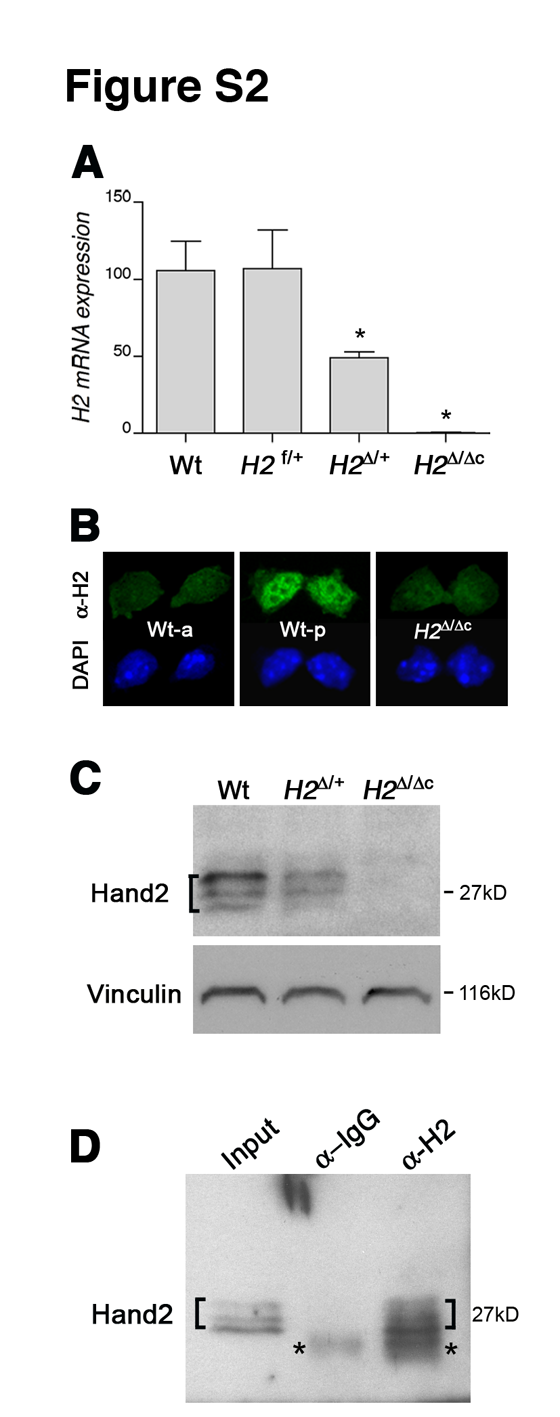

Supplement: Figure S2 — Clearance of Hand2 transcripts from mutant forelimb buds and specificity of α-Hand2 antibodies. (A) Q-PCR analysis to determine Hand2 transcript levels in wild-type, Hand2 floxed (H2 f), Hand2 heterozygous and Hand2 deficient limb buds at E10.25–10.5 (33–35 somites; n = 6–8). Note that no Hand2 transcripts are detected in Hand2 deficient limb buds. Bars: ±standard deviation. asterisk: P = 0.0009. (B) Immunofluorescense using α-Hand2 antibodies (M-19, Santa Cruz) reveals the specific nuclear localization of Hand2 proteins in posterior (Wt-p) but not anterior (Wt-a) limb buds mesenchymal cells. No specific fluorescence is detected in mesenchymal cells isolated from Hand2 deficient limb buds. (C) Hand2 proteins are cleared from Hand2 deficient limb buds by embryonic day E10.5. Protein extracts were normalized for their vinculin content. (D) Immunoprecipitation (IPP) of Hand2 proteins from E11.0 limb buds. Hand2 proteins are detected by Western blotting. Control: α-IgG. Asterisks indicate the cross-reactivity with the light chains of the IgGs (control and α-Hand2) used for IPP. (2.42 MB TIF) [file pgen.1000901.s002.tif]

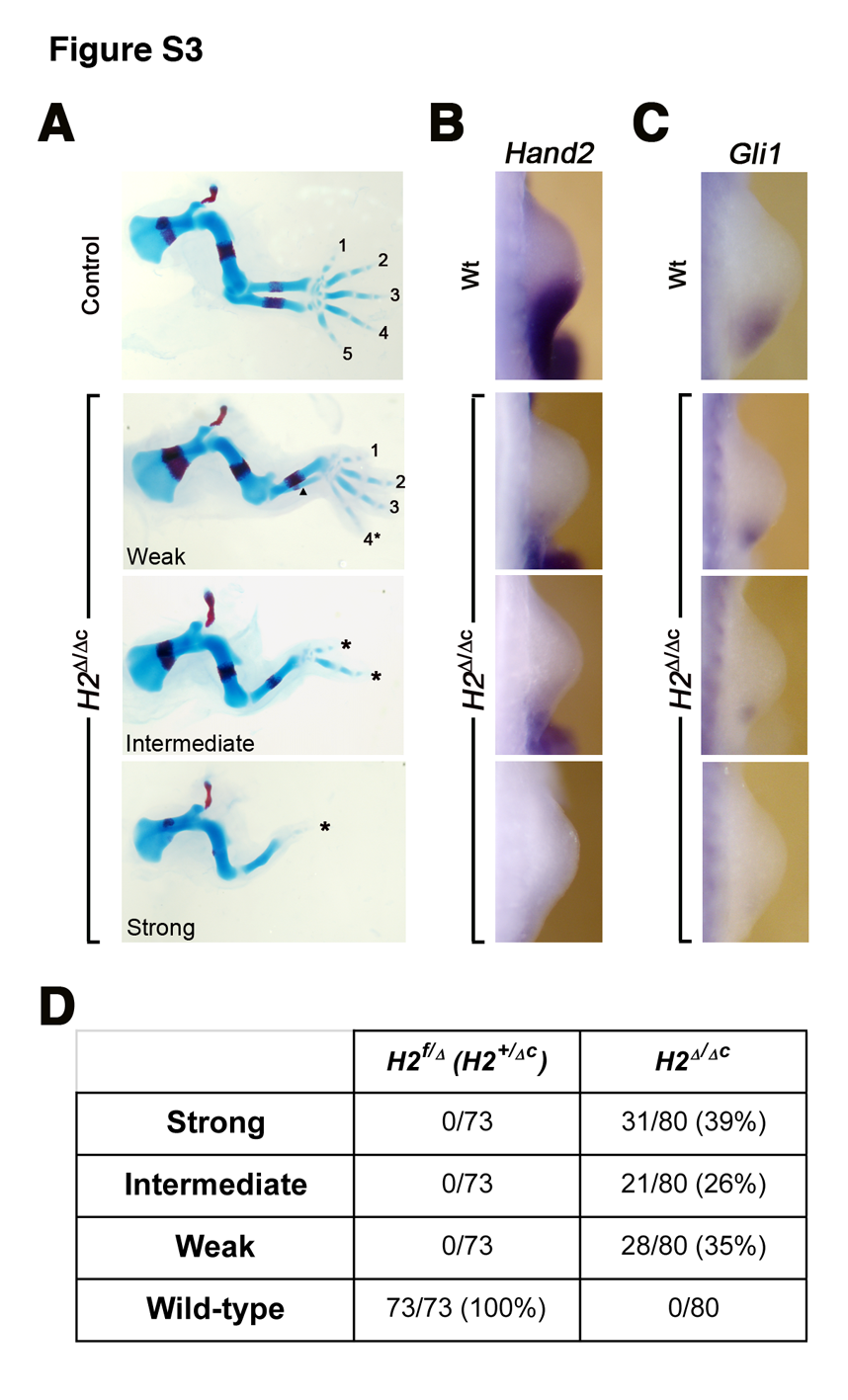

Supplement: Figure S3 — Incomplete/delayed inactivation of Hand2 in forelimb buds results in a hypomorphic phenotype. (A) Skeletal preparations of control (Prx1-Cre heterozygous) and Hand2 deficient forelimbs at E14.5. Due to slight variability in Prx1-Cre mediated inactivation of the conditional Hand2 allele in forelimb buds, three classes of skeletal phenotypes are observed. The most hypomorphic phenotype (Weak) results in formation of two misplaced zeugopodal bones, three anterior digits and a hypoplastic digit that resembles digit 4 (indicated by an asterisk). The arrowhead points to the twisted bones of the zeugopod. The less hypomorphic phenotype (Intermediate) results in formation of one zeugopodal bone and two digits. The null phenotype (Strong) is identical to the skeletal phenotypes observed in Shh deficient limb buds (Figure 1A). Asterisks indicate digits with unclear identities. (B) Analysis of Hand2 expression reveals the variable nature of Prx1-Cre mediated inactivation of Hand2 at E9.75 (28 somites). (C) This variability is also apparent when levels of SHH signal transduction are monitored by Gli1 expression at E9.75 (27 somites). Complete absence of Hand2 (B) and Gli1 transcripts (C) was observed in 50% of all Prx1-Cre1, Hand2 deficient limb buds (n = 4/8). The others display varying degrees of Hand2 and Gli1 expression. All limb buds are oriented with the anterior to the top and the posterior to the bottom. (D) Table summarizing the frequencies of the three classes of limb skeletal phenotypes observed in Hand2 mutant forelimbs. This variability is in agreement with the fact, that developmentally slightly later Hand2 inactivation in hindlimb buds results in almost normal Shh expression and limb skeletal development (Figure 2). Taken together, these results indicate that Hand2 needs to be inactivated very early and rapidly during the onset of limb bud development to disrupt establishment of the posterior Shh expression domain. (3.68 MB TIF) [file pgen.1000901.s003.tif]

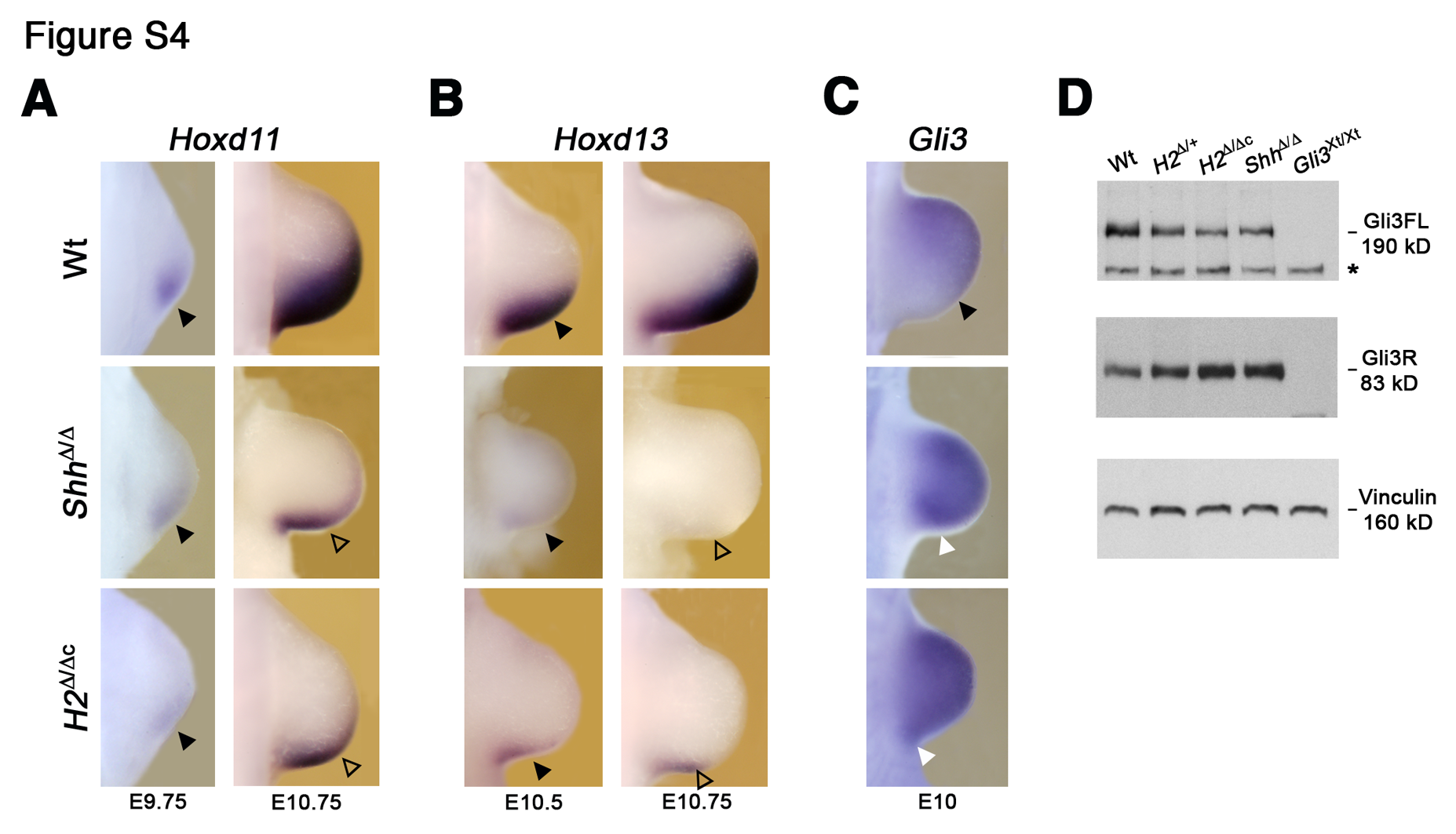

Supplement: Figure S4 — Activation of 5′HoxD genes and posterior expansion of Gli3 expression in Hand2 deficient limb buds. Hoxd11 expression at E9.75 (27 somites) and E10.75 (36 somites). Expression of Hoxd11 is initiated in limb buds lacking Hand2 (arrowheads), but its up-regulation is disrupted. (B) Hoxd13 expression is initiated, but rapidly down-regulated in Hand2 deficient limb buds (arrowheads E10.5, 33 somites). (C) Gli3 expression is expanded posteriorly in Hand2 deficient limb buds at E10.0 (32 somites; compare white to black arrowhead). In Shh deficient limb buds, Gli3 is not expanded to the posterior margin (compare white to open arrowheads). All limb buds are oriented with the anterior to the top and the posterior to the bottom. (D) Inactivation of Hand2 alters Gli3 protein processing. Protein extracts prepared from limb buds of the indicated genotypes at E10.5 (35 somites) were analyzed by immunoblotting using α-Gli3 antibodies. The full-length Gli3 protein is about 190 kD, while the processed Gli3R isoform is about 83 kD. Note that Gli3R form is more abundant in Hand2 and Shh deficient than in wild-type limb buds. Samples are normalized for their vinculin contents. The asterisk points to an unrelated cross-reacting protein. (5.95 MB TIF) [file pgen.1000901.s004.tif]

## Figure S5

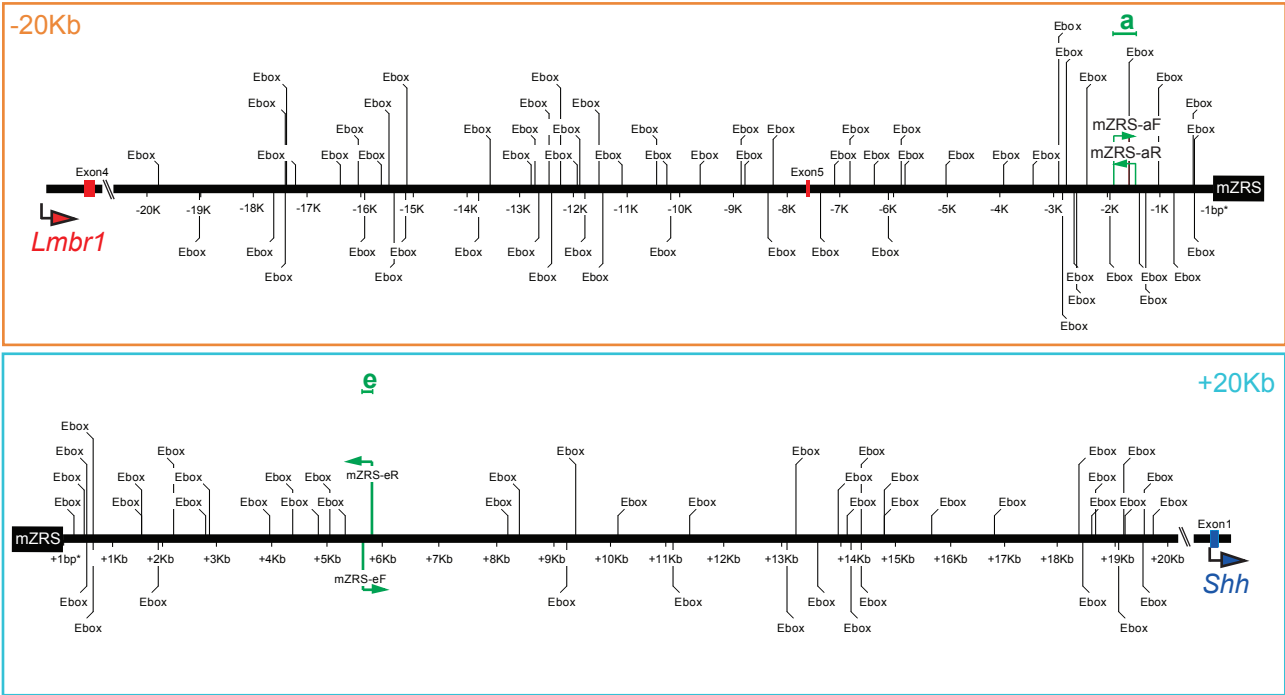

Supplement: Figure S5 — The genomic landscape encompassing the mouse ZRS. Scheme depicting part of mouse chromosome 5 (Ensemble: Mus musculus genomic region from position 29621310 to 29662806) analyzed in the ChIP experiments by Q-PCR. The Lmbr1 locus encodes the mouse ZRS (1.67 kb) within intron 4, which is about 800 kb away from the Shh locus. The 6 Ebox elements (1 to 6) located in the ZRS are indicated. The framed orange and blue boxes indicate the 20 kb downstream and upstream flanking regions. These two regions are shown in the enlargements and potential Ebox elements are indicated. Coding exons are represented by filled boxes. Amplicon a is located about 2 kb downstream and amplicon e about 6 kb upstream of the ZRS (the primers used for Q-PCR amplification are indicated by green arrows). (0.32 MB PDF) [file pgen.1000901.s005.pdf]

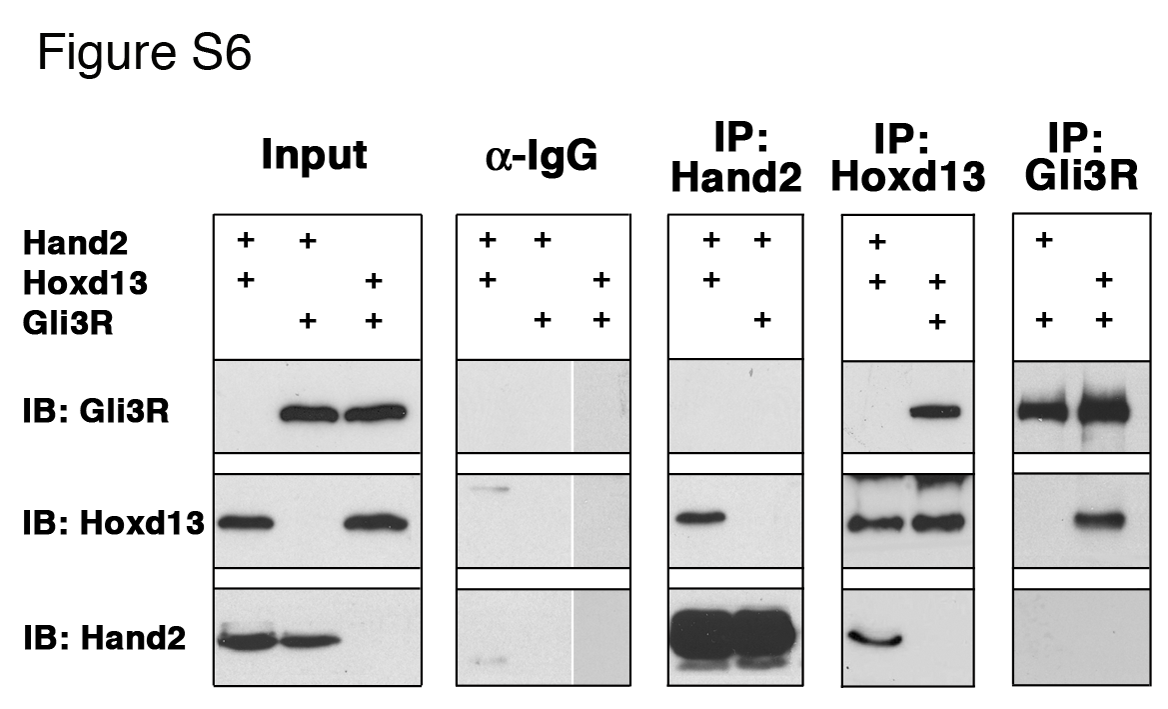

Supplement: Figure S6 — Evidence that Hand2 interacts directly with the Hoxd13 but not Gli3R protein. Co-immunoprecipitation reveals the direct interaction of Hand2 with Hoxd13 in HEK293T cells (Hand2: Flag-epitope tagged; Gli3R: Myc-epitope tagged). In contrast, Gli3R is unable to directly interact with Hand2, but binds to Hoxd13 [12]. Protein extracts were immunoprecipitated (IP) using the following antibodies: α-Flag for Hand2, α-Hoxd13 for Hoxd13, α-Myc for Gli3R and immunoblotted (IB) using the appropriate antibodies. (2.52 MB TIF) [file pgen.1000901.s006.tif]

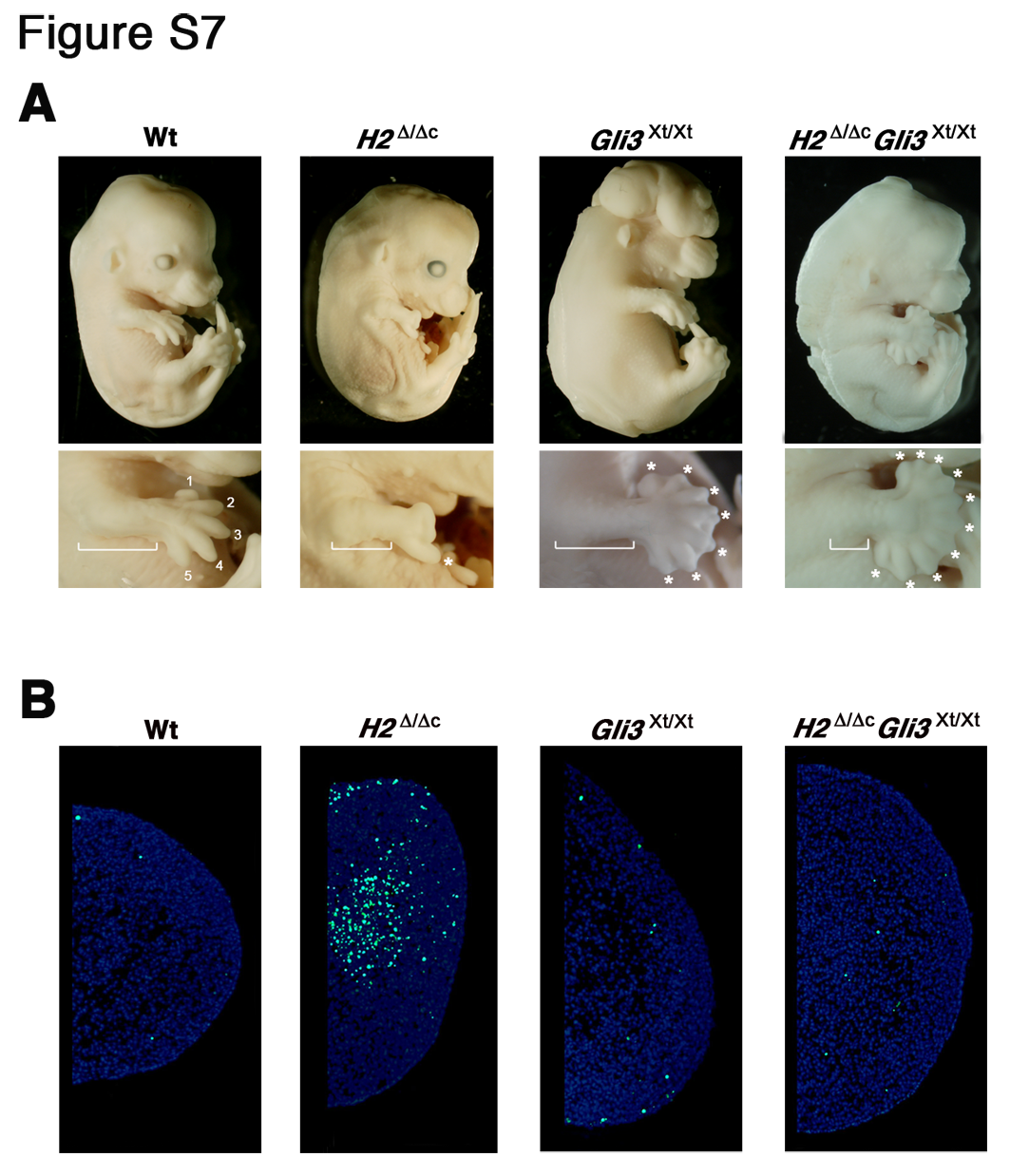

Supplement: Figure S7 — Morphological defects in limb buds lacking Hand2 and Gli3. (A) The forelimb morphology of double mutant mouse embryos at E14.5. Note the stunted forelimbs and the extreme pre- and post-axial polydactyly in comparison to Gli3 Xt/Xt limb buds. White brackets indicate forelimb length. Asterisks indicate digits with undetermined identities. (B) The massive apoptosis of mesenchymal cells in Hand2 deficient limb buds is suppressed in limb buds lacking both Hand2 and Gli3. Apoptotic cells were detected by TUNEL fluorescence on limb bud sections at E10.25 (33 somites). Sections are oriented with the anterior to the top and posterior to the bottom. (3.99 MB TIF) [file pgen.1000901.s007.tif]
