# Supplementary material for: Distinct Roles of Hand2 in Initiating Polarity and Posterior Shh Expression during the Onset of Mouse Limb Bud Development
Source: PLoS Genet. 2010 Apr 8;6(4):e1000901. doi: 10.1371/journal.pgen.1000901 (PMC2851570; doi:10.1371/journal.pgen.1000901)
Supplement: Table S1 — Oligos used for the study. All primers used for genotyping of mice and embryos, Q-PCR analysis of Hand2 transcripts, Q-PCR analysis of the ChIP experiments are listed. Conditions for use are available upon request. (0.04 MB DOC) [file pgen.1000901.s008.doc]

**Table S1**

| **Allele** | **Forward primer** | **Reverse primer** |
| --- | --- | --- |
| *H2* wt/floxed | 5’-CTGTGCCTGGTGCTTCGTTTTGTG-3’ | 5’-CCCTCCTCCACCACCACTGCTCAT-3’ |
| *H2* floxedneo | 5’-CTGTGCCTGGTGCTTCGTTTTGTG-3’ | 5’-CAGGACATAGCGTTGGCTACCCG-3’ |
| *H2* null | 5’-CCTCGGCAATTAGCAACGTGAACATC-3’ | 5’-CCCTCCTCCACCACCACTGCTCAT-3’ |

| **cDNA** | **Forward primer** | **Reverse primer** |
| --- | --- | --- |
| *Hand2* | 5’- AAGAGGAAGAAAGAGCTGAATGAGAT-3 | 5’- CGTTGCTGCTCACTGTGCTT-3’ |

| **ChIP amplicon** | **Forward primer** | **Reverse primer** |
| --- | --- | --- |
| **a** | 5’- TTCGTTTGATGACTAAATGAGGTAAT -3 | 5’- tctccttataaattgcaggtctaaaaa -3’ |
| **b** | 5’- tggcatgagagagttagtggtc -3 | 5’- tcacagcactgtgttctcctc -3’ |
| **c** | 5'- GTCACAGTTTGAGATTGTCCTGGT -3' | 5'- tgaaagaatccaatgaacgctcatg -3' |
| **d** | 5'- gcacatctggaatgcatgcagg -3' | 5'- gcttaagtttgagtttaagtcacaatc -3' |
| **e** | 5’- CCAAAGGCTCTAGGTTGCTG -3 | 5’- gcccttcccactaatcttcc -3’ |
| mouse β-actin | 5'- ACACTGTGCCCATCTACGAGG -3' | 5'- CGCTCGTTGCCAATAGTGATG -3' |
