# Supplementary material for: Distinct Roles of Hand2 in Initiating Polarity and Posterior Shh Expression during the Onset of Mouse Limb Bud Development
Source: PLoS Genet. 2010 Apr 8;6(4):e1000901. doi: 10.1371/journal.pgen.1000901 (PMC2851570; doi:10.1371/journal.pgen.1000901)
Supplement: Text S1 — Supporting materials and methods. (0.08 MB DOC) [file pgen.1000901.s009.doc]

**Supporting** **Materials and Methods**

**Generation of *Hand2* conditional mutant mice.** The *Hand2* genomic locus was modified to introduce a *loxP* and *EcoRV* site into the *NarI* site 5’ of exon1. The second *loxP* site was inserted into the *BamHI* site 3’ of exon2 together with the selection cassette containing the pGK-*Neo* gene flanked by two *FRT* sites. The resulting *Hand2* targeting vector (Figure S1A) was linearized with *XhoI* and introduced into mouse R1-ES cells. G418 resistant ES-cell clones (576 in total) were screened by Southern blot analysis as shown in Figure S1B. One ES-cell clone (4D7) was fully recombined and germ-line transmission from chimeric mice was obtained. The *Hand2* floxed (*H2*^f^) and null (*H2*^Δ^) alleles were obtained by crossing to FLPe [1] and CMV-Cre mice [2]. *Hand2* was inactivated specifically in the early limb field mesenchyme (*H2*^Δ/Δc^) by crossing *H2*^f/f^ females with males heterozygous for the *H2*^Δ^ allele and the *Prx1-Cre* transgene [3].

**Mice and embryos.** Double mutant embryos lacking *Hand2* and *Gli3* in their limb buds were generated by mating *H2*^f/f^*Gli3*^Xt/+^ females with *H2*^Δ/+^*Gli3*^Xt/+^*Prx1-Cre*^tg/+^ males. For this analysis the *Xt^J^* allele of the spontaneous *Gli3* loss-of-function mutations was used, as it is best characterized [4]. *H2*^ΔΔc^*Gli3*^Xt/Xt^ limbs do not display significant variations in their limb bud skeletal phenotypes in contrast to *H2*^ΔΔc^ single mutant limbs. Mice and embryos were genotyped by PCR using DNA isolated from tail clips, head tissue and/or extra-embryonic membranes. The primers used for genotyping the *Hand2* alleles are listed in Table S1. Noon of the day of vaginal plug detection was considered as embryonic day 0.5 (E0.5). Embryos were age-matched according to somite numbers.

**Cell death analysis.** Apoptotic cells were detected in whole limb buds and embryos by LysoTracker Red DND-99 (LysoT, Molecular Probes) or on sections using the *in situ* TUNEL kit (Roche). Sections were counterstained with Hoechst.

**Whole-mount *in situ* hybridization and skeletal preparations.** Whole-mount mRNA *in situ* hybridizations using digoxigenin-labelled antisense riboprobes were performed as described [5]. Embryonic skeletons were visualized by staining ossified bone tissue with Alizarin red and the cartilage with Alcian blue [6].

**Quantitative real-time PCR (Q-PCR) analysis.** Forelimb buds were collected at E10.0-10.5 (30-37 somites) and stored in RNA*later* (Ambion). Total RNA was extracted using the RNeasy kit (Qiagen) and cDNAs synthesized using Superscript III reverse transcriptase (Invitrogen). Transcript levels were quantified by Q-PCR using the ABI Prism 7000 real time PCR machine in combination with the Power SYBR Green PCR Master Mix (Applied Biosystems). Relative transcript levels were normalized to mouse *ribosomal protein L19*. Each result represents the mean of 6-8 independent samples per genotype (± standard deviation). The primers used to detect *Hand2* transcripts are shown in Table S1. The primer pairs for all other transcripts are identical to the ones used previously by Benazet et al. [7].

**Western blot and immunofluorescence analysis.** For western blots, mouse limb buds from E9.75-10.5 were dissected into PBS and lysed in RIPA buffer. Following centrifugation, supernatants were collected and the protein concentration determined using BCA (Pierce). Total proteins were used to detect Hand2 (40mg protein) and GLI3 (10mg) using anti-Hand2 (M-19, Santa Cruz) and affinity-purified rabbit anti-Gli3 antibodies, respectively. Samples were normalized by their vinculin content (Sigma). For immunofluorescence limb bud cells were dissociated in 0.5% Trypsin-EDTA for 10min at 37ºC and plated on coverslips for 5 hours. Cells were then fixed and processed for immunofluorescence. Cells were counterstained with Hoechst.

**Expression constructs.** The ZRS-luciferase (ZRS-Luc) reporter construct was generated as follows: the ZRS (*HindIII*-*HindIII* fragment of 1.67kb) [8] was amplified by PCR and cloned into a modified version of the pGL4 vector (Promega). One predicted *Ebox* site in the pGL4 vector backbone was mutated using the QuickChange II Site-Directed Mutagenesis Kit (Stratagene) and an 80bp fragment containing an adenovirus minimal promoter (80bp) was inserted into pGL4 and all critical regions sequenced. *In silico* analysis failed to reveal any canonical and/or non-canonical Gli binding sites in the ~1.7kb mouse ZRS sequence as previously reported [9]. The potential of Hand2 to directly trans-activate expression via the proximal *Shh* promoter was also assayed, but no Hand2-dependent luciferase activity was detected (data not shown). The Hand2 ORF was cloned into pcDNA3 and a FLAG epitope tag was inserted into the *PflMI* site at position 11 of the open reading frame. The Hoxd13 and Gli3R expression constructs were described previously [10,11].

**Co-immunoprecipitation experiments in cells.** Transfected HEK293T cells were collected after 24-28 hrs and incubated in lysis buffer (HEPES 10mM pH7.5, NaCl 150mM, EDTA 1mM, NP40 0.5%, protease inhibitors) for 30 min on ice. The cleared supernatants were incubated with protein G magnetic beads (Dynabeads, Invitrogen) coated with α-Flag M2 (Sigma), α-Myc (ATCC, clone 9E10) or α-Hoxd13 antibodies [12] overnight at 4°C. The immunocomplexes were washed in lysis buffer containing 0.1% Tween-20, separated by SDS-PAGE and transferred to polyvinylidene difluoride membranes (Millipore). One tenth of the protein extracts was loaded as input control.

**Supporting References**

1. Rodriguez CI, Buchholz F, Galloway J, Sequerra R, Kasper J, et al. (2000) High-efficiency deleter mice show that FLPe is an alternative to Cre-loxP. Nat Genet 25: 139-140.

2. Schwenk F, Baron U, Rajewsky K (1995) A cre-transgenic mouse strain for the ubiquitous deletion of loxP-flanked gene segments including deletion in germ cells. Nucleic Acids Res 23: 5080-5081.

3. Logan M, Martin JF, Nagy A, Lobe C, Olson EN, et al. (2002) Expression of Cre recombinase in the developing mouse limb bud driven by a Prxl enhancer. Genesis 33: 77-80.

4. Buscher D, Ruther U (1998) The XtJ allele generates a Gli3 fusion transcript. Mamm Genome 9.

5. Panman L, Galli A, Lagarde N, Michos O, Soete G, et al. (2006) Differential regulation of gene expression in the digit forming area of the mouse limb bud by SHH and gremlin 1/FGF-mediated epithelial-mesenchymal signalling. Development 133: 3419-3428.

6. Zuniga A, Zeller R (1999) Gli3 (Xt) and formin (ld) participate in the positioning of the polarising region and control of posterior limb-bud identity. Development 126: 13-21.

7. Benazet JD, Bischofberger M, Tiecke E, Goncalves A, Martin JF, et al. (2009) A self-regulatory system of interlinked signaling feedback loops controls mouse limb patterning. Science 323: 1050-1053.

8. Lettice LA, Heaney SJ, Purdie LA, Li L, de Beer P, et al. (2003) A long-range Shh enhancer regulates expression in the developing limb and fin and is associated with preaxial polydactyly. Hum Mol Genet 12: 1725-1735.

9. Vokes SA, Ji H, Wong WH, McMahon AP (2008) A genome-scale analysis of the cis-regulatory circuitry underlying sonic hedgehog-mediated patterning of the mammalian limb. Genes Dev 22: 2651-2663.

10. Ulloa F, Itasaki N, Briscoe J (2007) Inhibitory Gli3 activity negatively regulates Wnt/beta-catenin signaling. Curr Biol 17: 545-550.

11. Williams TM, Williams ME, Heaton JH, Gelehrter TD, Innis JW (2005) Group 13 HOX proteins interact with the MH2 domain of R-Smads and modulate Smad transcriptional activation functions independent of HOX DNA-binding capability. Nucleic Acids Res 33: 4475-4484.

12. Chen Y, Knezevic V, Ervin V, Hutson R, Ward Y, et al. (2004) Direct interaction with Hoxd proteins reverses Gli3-repressor function to promote digit formation downstream of Shh. Development 131: 2339-2347.

13. Srivastava D, Thomas T, Lin Q, Kirby ML, Brown D, et al. (1997) Regulation of cardiac mesodermal and neural crest development by the bHLH transcription factor dHAND. Nature Genetics 16: 154-160.
